# Zn-Salphen Acrylic Films Powered by Aggregation-Induced Enhanced Emission for Sensing Applications

**DOI:** 10.1007/s10895-023-03399-6

**Published:** 2023-09-04

**Authors:** Ernesto Enríquez-Palacios, Ana Victoria Robledo-Patiño, Gustavo A. Zelada-Guillén

**Affiliations:** School of Chemistry, National Autonomous University of Mexico (UNAM), Circuito Escolar s/n, Ciudad Universitaria, Mexico City, 04510 Mexico

**Keywords:** Fluorescent films, Zn-salphen complex, Aggregation-induced emission, Polymer sensor

## Abstract

Zn(II) complexes possess attractive characteristics for supramolecular chemistry, catalysis, and optoelectronic applications, while Zn-Salphen counterparts are also suitable as chemical sensors, although limited by solution-based to date. In this study, we report the synthesis of new polymers from methyl methacrylate, *n*-butyl acrylate, and a non-symmetrical Zn-Salphen complex. We show that this low-fluorescent complex exhibits aggregation-induced emission enhancement (AIEE) properties and that, the incorporation of AIEE complexes into a polymeric matrix make it possible to achieve fluorescent films with enhanced fluorescence suitable for sensing applications. As a proof of concept, these films could detect acetic acid, showing a decrease of up to 73% in the original fluorescence. Host/guest studies showed a subtle disruption of the emission in aggregates upon treatment with anion guests. These results indicate that an interaction between the guest and Zn-Salphen complex may occur, stabilizing or destabilizing the complex and causing a concomitant increase or decrease in emission.

**Supplementary Information:**

The online version contains supplementary material available at 10.1007/s10895-023-03399-6.

## Introduction

Metal tetracoordinate Schiff-bases generated by condensation of salicylaldehydes and 1,2-phenylendiamines, also called Metal-Salphen complexes, have interesting﻿ properties useful for multiple applications ranging from solar cells [1], fluorescent sensors [2, 3], optoelectronics [4, 5], supramolecular chemistry [6–9]. The versatility of Salphen complexes has also reached the field of new materials, such as metal–organic frameworks [10], and it is not surprising to find them even in biological applications [11, 12].

Tetra-coordination plays a critical role in the structure of the complex, providing a highly conjugated structure assisted by the phenyl ring bridging the iminophenyl groups. The Zn center provides rigidity and a coordination site for extra ligands, attracting attention in catalysis [13, 14]. In this sense, the geometry also facilitates intermolecular Lewis acid–base interaction [15, 16]. The unsaturated coordination sphere of the metal can be occupied by Lewis bases or other Zn-Salphen units by Zn···O interaction [17, 18], leading to self-assembly structures that are highly attractive in the supramolecular chemistry field [19, 20]. In addition, to study the behavior of metal complexes in a polymeric architecture, Zn-Salphen has been polymerized in different ways, from polycondensation between diamines and bis-salicylaldehydes to metal-catalyzed cross-coupling [21]. In many cases, highly ordered structures have been obtained. This approach is suitable, for example, for introducing binding sites for catalytic applications or generating chiral materials with tunable optical properties [22, 23].

The photoluminescent properties of Zn(II) complexes have been extensively studied [2, 4, 23, 24]. The diversity of ligands is undoubtedly responsible for the differences in photophysical behavior. In particular, Zn-Salphen complexes present interesting luminescent properties, such as fluorescence and electroluminescence for applications in ion sensing [2], OLEDs [3], etc. It is well known that the photoluminescence of these complexes is generally due to transitions from the highly delocalized π system in the organic framework [25]. Spectroscopic studies in solution have demonstrated the consequences of aggregates in solution, which in most cases produces a decrease in the fluorescence of many molecules. However, the structure plays a determinant role in the path of deactivation after aggregate formation [26, 27]. In many cases, for highly conjugated π systems, π-π stacking is involved in fluorescence quenching. It is important to emphasize that different interactions between aggregates are possible thanks to the planar geometry and the axial coordination with the metal center. These interactions are responsible, in other cases, for the enhancement of emission [24, 28].

Aggregation-induced enhanced emission (AIEE) is a phenomenon of great interest in the field of fluorescent molecules, derived from aggregation-induced emission (AIE) [29]. Contrary to most fluorophores, whose emission is quenched by concentration, the increase in fluorescence promoted by aggregation is less common in molecules. This phenomenon provides an important tool for multiple applications in the solid state [30]. The mechanism of this phenomenon has been well studied, and the increase or turn-on of the emission has been assigned in some cases to the restricted intramolecular motions (RIM) in molecules whose structure is capable of dissipating the energy of the excited state in solution, e.g., by rotation of phenyl or heterocyclic rings. However, in aggregates, these same rotations are sterically hindered.

AIEE has also been reported in Zn complexes [24, 28] and macrocyclic disalphens [31]. However, it has never been reported in mononuclear non-symmetrical Zn-Salphen complexes. Furthermore, the copolymerization of complexes with acrylic monomers to generate thin films to study host–guest interactions or use as sensors in the solid state seems to be a reasonable proof of concept. Recently, we have studied the incorporation of metal-Salphen complexes (Ni, Cu, and Zn) into a polymer matrix by radical polymerization with acrylic monomers to generate materials with binding sites available to coordinate anions [32–34]. It was found that the Zn-Salphen complex acts as a recognition unit for different guests, producing differentiated electrochemical responses against fluoride or acetate, depending on the nature of the guest and the supramolecular environment afforded synergistically by an acrylic polymer chain. However, to date, there are no reports of solid-state fluorescent sensors based on acrylic matrices and Zn-Salphen receptors. This is a significant gap in the literature, as such sensors would be highly desirable for the detection of metabolites of biological relevance in non-communicable diseases (NCDs), such as acetic acid. Acetic acid is a weak organic acid that is naturally produced in the body. It is also found in a variety of foods and beverages, such as vinegar, wine, and beer. Acetic acid can also be produced by bacteria that live in the mouth and gut. In NCDs, such as diabetes, cancer, and heart disease, the levels of acetic acid in the body can be altered. For example, people with diabetes often have higher levels of acetic acid in their blood. This is because the body produces more acetic acid when it breaks down glucose for energy. Therefore, the detection of acetic acid in NCDs can be used as a biomarker for these diseases. The development of solid-state fluorescent sensors based on acrylic matrices and Zn-Salphen receptors would be a significant advance in the field of biosensing. Such sensors would be portable, easy to use, and sensitive to low levels of acetic acid. They could be used to diagnose NCDs at an early stage, which would improve patient outcomes.

## Results and Discussion

### Synthesis and Characterization

**Scheme 1:**
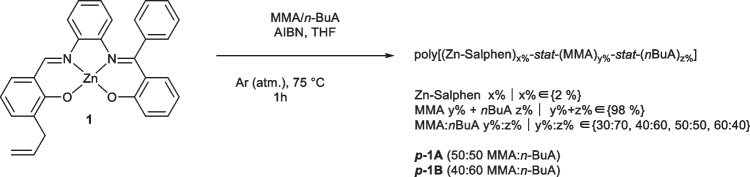
Synthesis of polymers ***p-1A*** and ***p-1B***

Complex **1** was prepared as described elsewhere by the condensation of (*E*)-2-(((2-aminophenyl)imino)(phenyl)methyl)phenol (ketimine) and 2-hydroxy-3-allyl-benzaldehyde and in situ coordination to Zn(OAc)_2_∙2H_2_O; the identity of the final product was confirmed by ^1^H-NMR and FTIR against the literature [34]. The polymers were synthesized by chemically initiated bulk polymerization using azobisisobutyronitrile (AIBN) (Scheme 1). Polymerization proceeded smoothly at 80 °C, yielding the polymers ***p-1A*** (50:50 MMA:*n*-BuA) and ***p-1B*** (40:60 MMA:*n*-BuA), in which complex **1** was present in 2% of the total mass and the acrylic components were at 98%. The monomer proportions used in this study were selected using a wider full factorial design of experiments carried out as a pre-screening process. The goal of such a preliminary screening of systems was to obtain a balance of stiffness, flexibility, and cohesiveness in films prepared from each formulation under a casting from solution approach, so as to discard those specimens with a suboptimal performance. In this regard, the monomers methyl methacrylate (MMA) and *n*-butyl acrylate (*n*-BuA) were varied in weight ratios ranging from 30:70 to 60:40 (i.e. 30:70, 40:60, 50:50, 60:40) so as to complete the rest of the composition apart from **1** (98%). The ratios MMA:*n*-BuA that afforded unviable films were those at 30:70 and 60:40, which were then discarded and are not further discussed in this work.

Polymers were characterized by gel permeation chromatography (GPC). Analysis showed high molecular weight (${\overline{{\varvec{M}}} }_{{\varvec{w}}}$) values for both polymers, ***p-1A*** (4.0 × 10^5^ g mol^−1^) and ***p-1B*** (3.3 × 10^5^ g mol^−1^), and a small decrease in dispersity (${\overline{{\varvec{M}}} }_{{\varvec{w}}}$/${\overline{{\varvec{M}}} }_{{\varvec{n}}}$) from 2.17 to 2.0 as the amount of MMA increased. Degrees of polymerization (DP) of 1665 (${\overline{{\varvec{X}}} }_{{\varvec{n}}}$) and 3622 (${\overline{{\varvec{X}}} }_{{\varvec{w}}}$) for ***p-1A*** and 1488 (${\overline{{\varvec{X}}} }_{{\varvec{n}}}$) and 2985 (${\overline{{\varvec{X}}} }_{{\varvec{w}}}$) for ***p-1B*** were estimated. Estimates of the average number of Zn-Salphen units ranged between 7.4–16.1 and 6.6–13.2 for ***p-1A*** and ***p-1B***, respectively. Interestingly, the polymers showed good stability up to 342 °C in the case of ***p-1A*** and 318 °C for ***p-1B*** (5% weight loss). Differential scanning calorimetry (DSC) showed glass transition temperatures of 21.3 °C for ***p-1A*** and 34.3 °C for ***p-1B***.

Polymers possessed suitable characteristics to form films easily by simple solvent evaporation. The solutions were prepared by dissolving 70 mg of the respective polymer in 1.2 mL of filtrated THF. The solutions were then rapidly spread onto a dust-free glass microscope slide (7.6 cm × 2.6 cm). The glass slide was left in a Petri dish until the solvent evaporated, and then dried at 80° C in a preheated oven for variable time. Finally, the membranes were immersed in de-ionized water and separated by pulling out from the glass slide. The thickness of the films was (89 ± 11) μm and (77 ± 14) μm for ***p-1A*** and ***p-1B***, respectively.

### Photophysical Properties of Zn-Salphen Complex

**Fig. 1:**
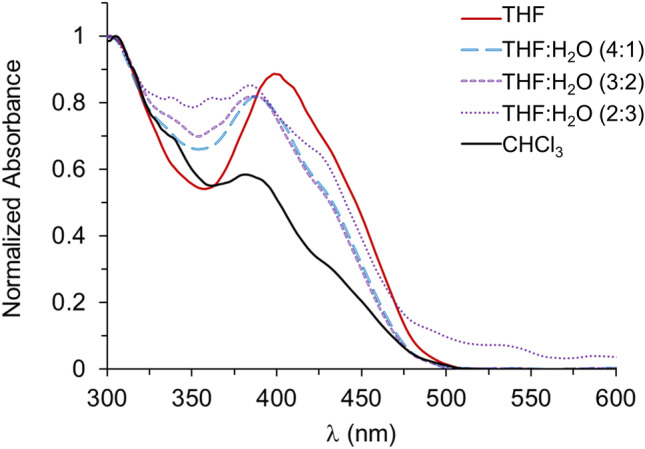
Normalized absorbance of complex **1** in chloroform and THF upon addition of fractional volumes of water up to 2:3 (THF:H_2_O). Final concentration of 5.04 × 10^–5^ M of **1** in all solutions

The absorption spectra of complex **1** were recorded in THF, CHCl_3_, and mixtures of THF:H_2_O (Fig. 1). As observed in coordinating solvents such as THF, two bands were identified, the absorption maxima at 300–304 nm, and an intense band at 398 nm. On the other hand, absorption of **1** in the less coordinative solvent CHCl_3_ results in a decrease in the absorption and a hypsochromic shift of the band from 398 to 382 nm, and a shoulder at *ca.* 430 nm. Absorption bands at 300 nm and 398 nm are assigned to the π-π* and n-π* intraligand transitions, respectively [9].

The trend of Zn-Salphen complexes to form aggregates in less coordinative solvents or at higher concentrations has been previously studied [18–23, 31]. Intermolecular Lewis acid–base interactions, *e.g.*, between the Zn center in one complex and O of another complex, have been proposed. Concomitant hypsochromic (*H* aggregates) or bathochromic (*J* aggregates) shift of fluorescent emission has been observed depending on the relative orientation between aggregates. Thus, the decrease in the absorption of the band at 398 nm suggests interaction by aggregates, and considering the blue-shifted band measured in chloroform, it can be hypothesized that *H* aggregates make a major contribution to the spectrum. Then, the spectrum of **1** in aggregates, induced by THF/water mixture, should be consistent with the formation of that kind of aggregates, which is the case for this complex, judging by the decrease in absorbance and hypsochromic shift of the band at the same zone.

**Fig. 2:**
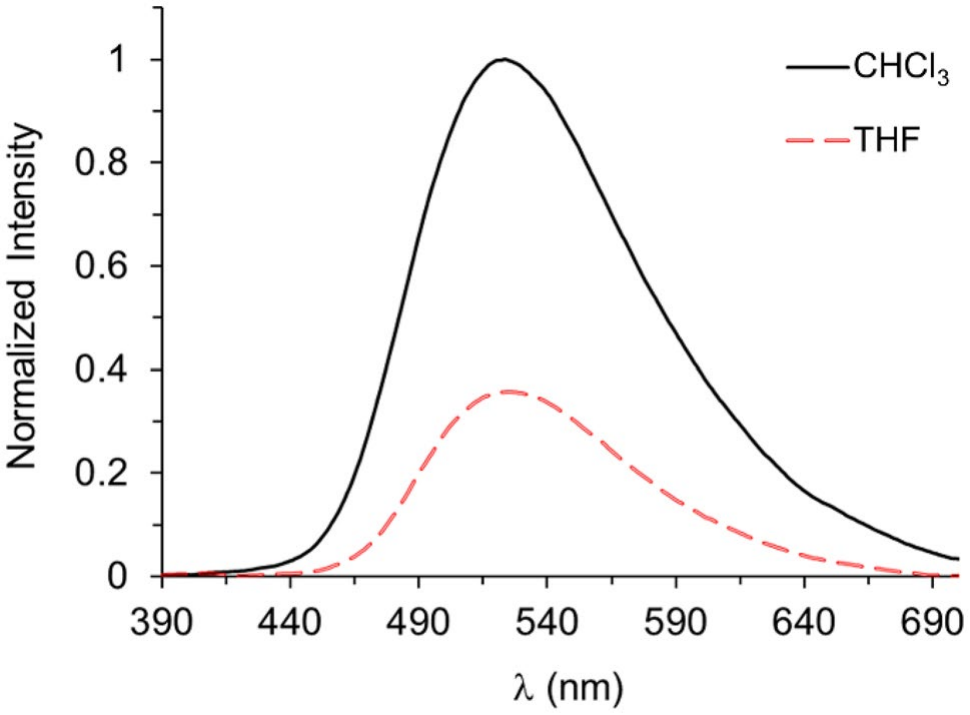
Native fluorescence (λ_exc_ = 365 nm) of Zn-Salphen complex dissolved in THF (dashed) and chloroform (solid) at the same concentration (5.04 × 10^–5^ M)

The Zn-Salphen complex used in this work was found to be low emissive in THF solution, although in chloroform it displays an increase in fluorescence of almost 3 times (Fig. 2). The fluorescent emission λ_max_ suffers minimal change, passing from 524 nm in THF to 523 nm in the less coordinative solvent chloroform, evidencing a poor solvatochromic effect. In this sense, many studies on Zn(II) complexes have pointed toward dimeric aggregates [9], intraligand charge transfer, or π-π stacking as the responsible mechanism for the fluorescence quenching [22]. In any case, it is evident that unlike those previous Zn complexes, less coordinative solvent promotes the formation of aggregates that are significantly more fluorescent than the de-aggregated form of complex **1**.

### AIEE Behavior of Complex 1

**Fig. 3:**
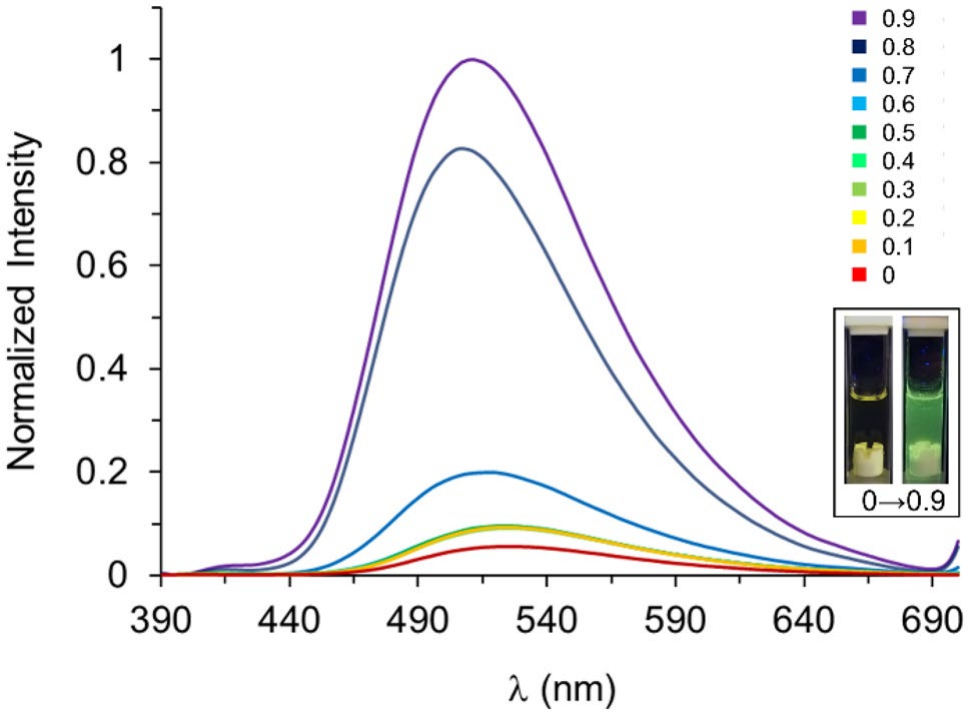
Normalized fluorescence (λ_exc_ = 365 nm) of complex **1** upon addition of fractional amounts of water. Final concentration of each solution was 5.04 × 10^–5^ M. Inset images: fluorescence of **1** in THF solution (left) and forming aggregates (right) upon irradiation with a 365 nm lamp

An aggregation-induced emission enhancement (AIEE) phenomenon was interestingly observed in the complex **1** in the solvent system THF/water (Fig. 3). These AIEE studies showed that increasing the fractional water content in a THF solution of complex **1** promotes the formation of aggregates, which in turn increase the fluorescence intensity. This enhancement in the fluorescence was observed in a first step upon adding 10% of water, then, a plateau was reached up to 60%. After 70% of water content, a slight increase was found again. Finally, the largest enhancement was measured at 80% of water content, and after 90% an increase in the original emission of about 18 times was observed.

The wavelength of the emission maxima drifted at different water contents. For example, between 0 and 60%, it oscillated from 522 to 526 nm. A stronger hypsochromic shift was observed at higher water contents. For example, at 70%, the λ_max_ (519 nm) is 5 nm shifted compared to complex **1** in THF (λ_max_ = 524 nm). Although stronger changes are observed from 80% (λ_max_ = 507 nm) and 90% (λ_max_ = 511 nm) of water content with 17 nm and 13 nm, respectively, in these solutions the effect on the emission maxima and intensity of the aggregates is more pronounced due to their high presence in the solution.

In accordance with similar studies in highly concentrated solutions [27], the changes in the UV–vis spectra and the behavior in fluorescence suggest that once the complex starts to aggregate due to insolubility, the intermolecular coordination (Zn∙∙∙O) and sterically demanding phenyl ring enable the formation of various aggregate species. Therefore, the fluorescent emission is principally related to the oligomeric aggregates. In addition, this result suggests that π-π stacking interaction is not sufficiently strong to deactivate the excited state upon aggregation. Hence, there is a mechanism for the enhancement of fluorescent emission dependent on the aggregate formation, as evidenced by the hypsochromic shift of the fluorescent and absorption maxima. This led us to consider the restricted intramolecular motion (RIM) [29] of the phenyl group attached to the imine as the driving force of the fluorescence enhancement. The results obtained in the AIEE experiment also suggest that the integration of complex **1** into a polymeric matrix would open the door to aggregation-induced enhanced emission in polymer-based optical sensors in either solution or the solid state, thus facilitating schemes of low-cost portable detection of suitable analytes.

### Photophysical Properties of Polymer

**Fig. 4:**
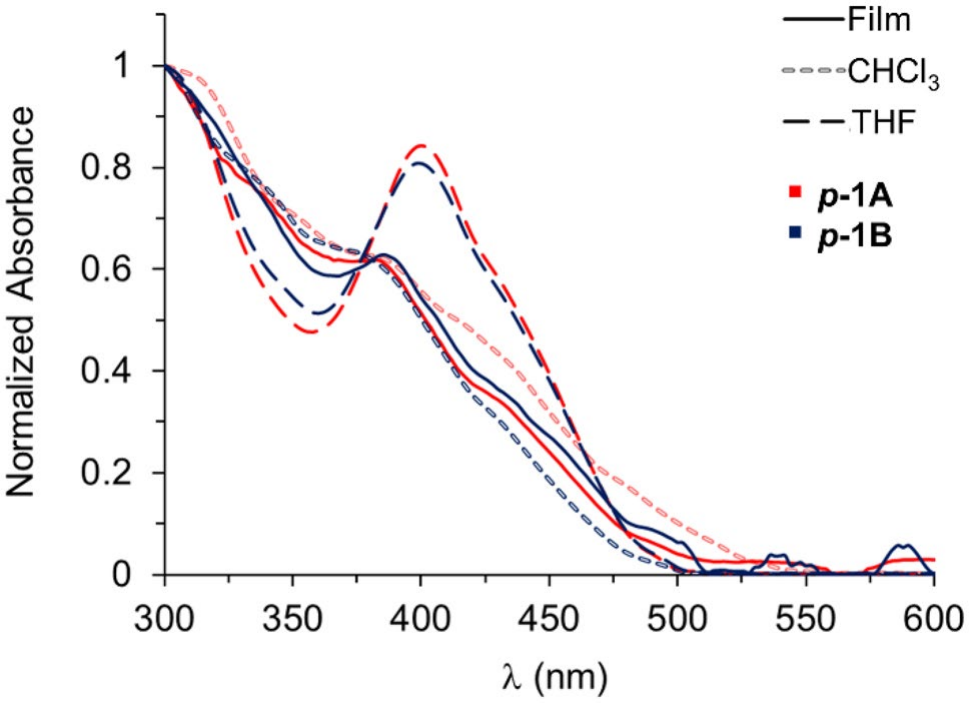
Normalized absorption spectra of Zn-Salphen polymers ***p-1A*** and ***p-1B*** in THF (dashed bold black), chloroform (dashed light gray) and film (solid bold). Concentration of 1 mg/mL of polymer at 2% nominal content of complex **1**

UV–visible spectra of the polymers were obtained in films and solutions (Fig. 4). The spectra of ***p-1A*** and ***p-1B*** obtained in THF show two principal bands, one with a maximum (λ_max_) at 300 nm and a low-energy absorption band at 400 nm. The spectra in films show an increasing absorption from approximately 480 nm to 290 nm and an apparent band at 382 nm and 386 nm for ***p-1A*** and ***p-1B***, respectively. This indicates that the local maximum at 400 nm undergoes a hypsochromic shift (14–18 nm) when moving from solution in THF to film. The UV–visible spectra of the polymers and complex **1** in THF remain practically unchanged, which shows that there is no interaction by aggregation. Analogous spectra were observed for the polymers in chloroform and films, suggesting that in the solid state, interactions by aggregates like those found in non-coordinating solvents can also occur.

**Fig. 5:**
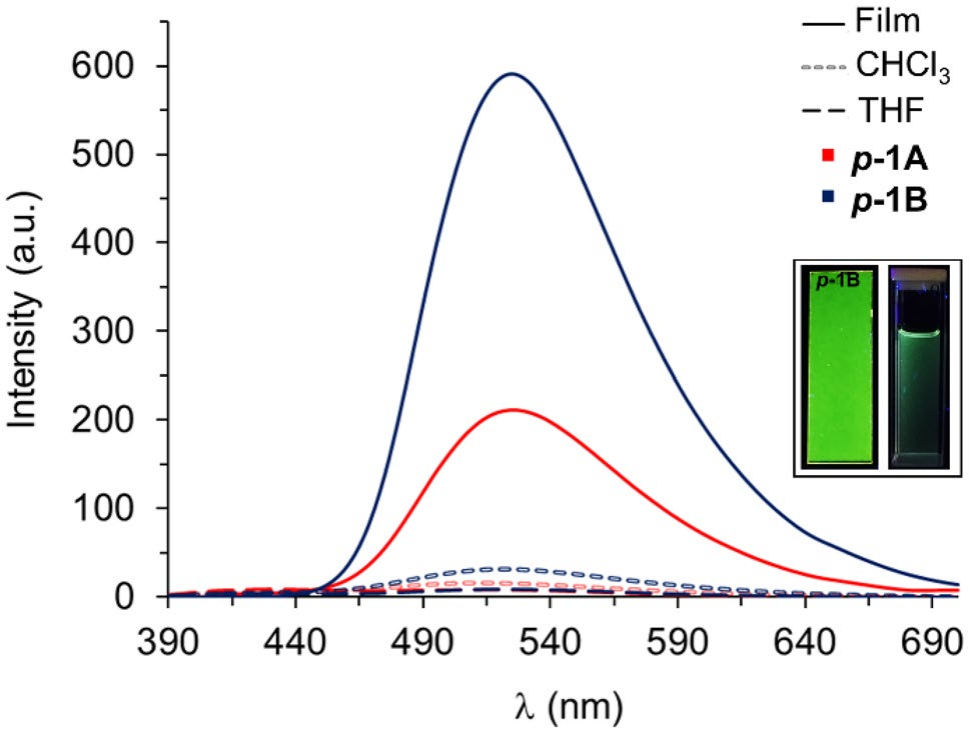
Fluorescent emission (λ_exc_ = 365 nm) of ***p-1A*** (red) and ***p-1B*** (blue) in THF (dashed bold black), chloroform (dashed light gray) and film immersed in water (solid bold). Concentration of polymer in solution of 0.5 mg/mL with 2% of nominal content of complex **1** in the polymer. Inset images: fluorescence of ***p-1B*** dissolved in chloroform (right) and casted in film (left) upon irradiation with a 365 nm lamp

Emission spectra of the polymers were recorded in solution and film (Fig. 5). The polymers in solution showed an intensity quite similar to that displayed by complex **1**. Once again, chloroform promoted the increase in fluorescence, likely due to aggregates. According to previous observations for complex **1** in solution, polymer ***p-1A*** has a fluorescent emission centered at 510 nm in THF, while in chloroform it increases to 512 nm. A strong shift of 16 nm was observed in film, with the emission maxima centered at 526 nm. On the other hand, ***p-1B*** showed a modest shift of the λ_max_ from THF (524 nm) to chloroform (522 nm), and finally, a small bathochromic shift of 1 nm was observed in film (λ_max_ = 525 nm).

Photoluminescence in the solid state exhibits an intense enhancement of as great as 38 times in the case of ***p-1A*** and 65 times for ***p-1B***, compared to their solutions in THF. Such a significant increase in fluorescence is consistent if we consider the aggregation-induced emission enhancement shown by complex **1**. In this sense, it can be argued that restricted intramolecular motion (RIM) of the Zn-Salphen unit in polymers could enhance the fluorescence in the solid state, given the low Zn-Salphen units (7.4–16.1) relative to the total chain size (1665–3622) for ***p-1A*** as an example.

### Sensing Properties

The supramolecular recognition of a copolymerized Zn-Salphen scaffold has been previously studied using non-kinetically monitored steady-state electrochemical techniques against different guests [34]. Such work showed that the binding of anions to the Zn-Salphen complex resulted in a change in the polymer’s charge, which could be measured using potentiometry. On the other hand, interaction of Lewis bases with the metal center is known to produce de-aggregation of self-assembled Zn-Salphen complexes [19, 26]. This phenomenon is due to the fact that Lewis bases can donate electrons to the metal center, which weakens the pre-existent intermolecular metal–ligand bonds (M‧‧‧O) and leads to the dissociation of the assemblies. Since fluorescent response is a characteristic of the aggregation state of the complex and, as shown in an earlier section, copolymer films ***p-1A*** and﻿ ***p-1B*** maintain a photophysical emission trend compatible to the behavior shown by aggregated molecular counterparts (Fig. 5), then it is reasonable to infer that this phenomenon could be exploited in polymer films for optical detectors under a turn-off fashion. In such a device, the addition of a suitable Lewis-basic guest would cause the preexisting Zn-Salphen conglomerate domains to de-aggregate, which would result in a negative change in the complex's fluorescent emission at a local level. This change in fluorescence could be used to detect the presence or absence of a suitable analyte in a simplified monitoring format that does not require electrochemical monitoring schemes. However, the capacity of certain guests to spontaneously displace proton interchange equilibria, such as those with Brønsted-Lowry weak base behavior, remains unclear. It is unknown whether this capacity would drive an eventual host–guest response positively or negatively.

**Fig. 6:**
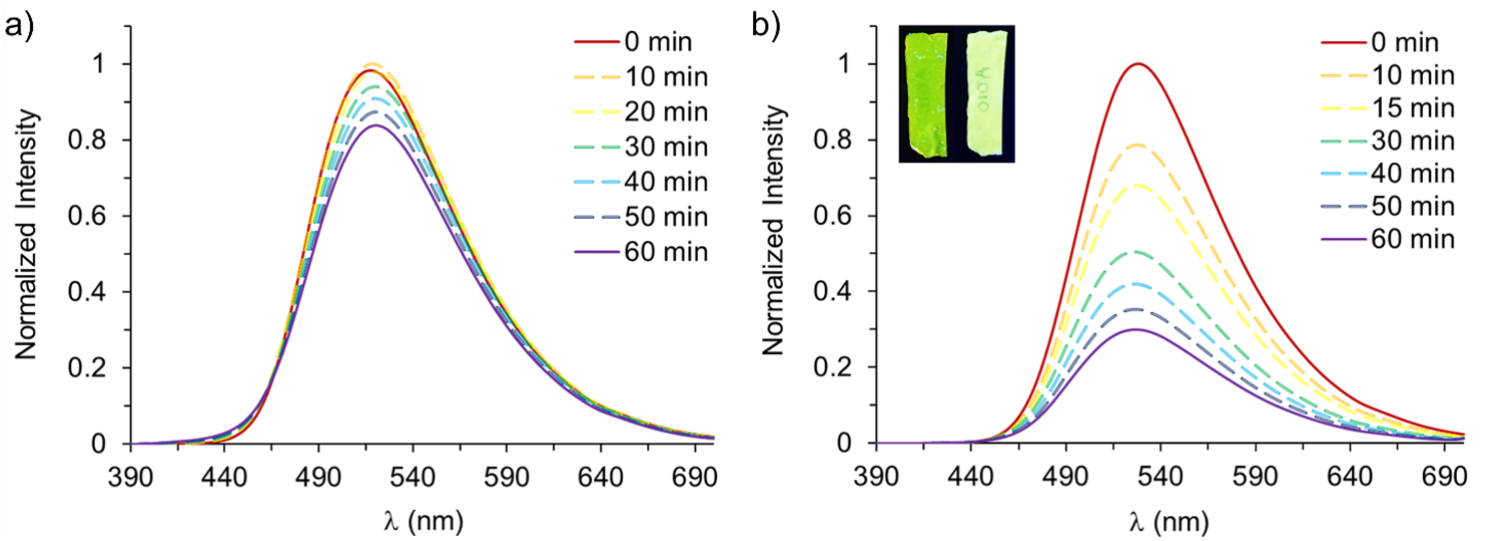
Normalized emission spectra (λ_exc_ = 365 nm) of film ***p-1A*** measured at different times in presence of F^−^ (**a**) and acetic acid (**b**) solutions. Concentration of solutions 10 mM. Inset images: fluorescence of a stripe of ***p-1A*** film before (left) and after treatment with acetic acid under UV light (365 nm)

**Fig. 7:**
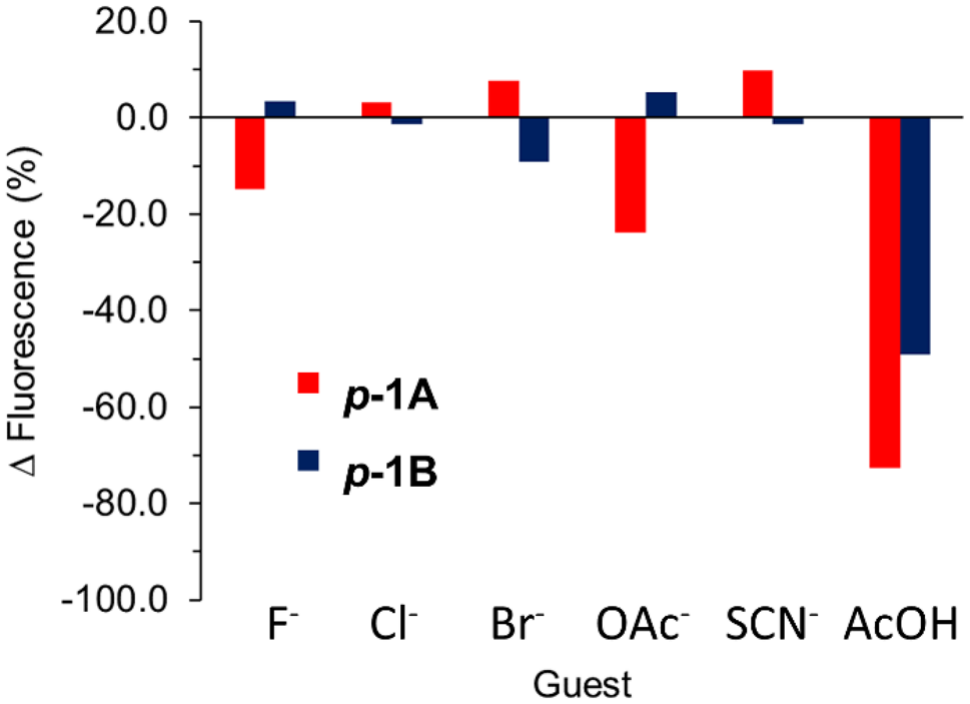
Fluorescence emission (λ_exc_ = 365 nm) performance of films ***p-1A*** and ***p-1B*** in presence of 10 mM solutions of guests and acetic acid

To address these questions, we evaluated the optical sensing properties of ***p-1A*** and ***p-1B*** films in the solid state. We immersed the films in 10 mM solutions of selected guests, including fluoride (F^−^), chloride (Cl^−^), bromide (Br^−^), acetate (OAc^−^), and thiocyanate (SCN^−^). The fluorescence emission spectra of the films were measured at different times for one hour (Figs. 6 and 7 and Supplementary Figs. S1–S10). We also tested the protonated counterpart of one of the earlier guests, acetic acid (AcOH), as a surrogate for a metabolically relevant derivative. This further clarified the remaining open questions.

In general, small changes in emission were observed for the anions throughout the time of each experiment. For example, in the case of the film ***p-1A***, a slight increase in fluorescence was observed against fluoride after 10 min, followed by a drop of about 15% of total emission (Figs. 6a and 7). Thiocyanate showed a similar trend, where an overall increase of almost 10% was achieved after 20 min, followed by a return to lower intensities under a semi-asymptotical fashion (Fig. S1). Chloride and bromide produced an enhancement of 3% and 8%, respectively (Figs. S2 and S3) after 10 to 20 min, whereas, on the contrary, acetate was the anion with the highest turn-off response (Fig. S4), producing a quench of 24% of the total fluorescence after 60 min. On the other hand, film ***p-1B*** displayed a modest response, although the effect was the inverse to that shown by the earlier counterpart. For this film, chloride, bromide, and thiocyanate were quenchers, with quenching efficiencies of 1%, 9%, and 1%, respectively (Figs. S5, S6 and S7). Fluoride and acetate showed a fluorescence enhancement of only 3% and 5%, respectively, after approximately 20 min (Figs. S8 and S9). This was followed by a return to more conservative values over the longer term. However, the results also suggest that the response and kinetics is related to the composition of the films. De-aggregation apparently occurs more easily for MMA:*n*-BuA ratios of 50:50 than 40:60. Therefore, acetate is the most promising candidate to show a proof-of-concept turn-off behavior in film ***p-1A***. We propose as well using the protonated counterpart of this anion AcOH to explore whether its incorporation would promote a differential change in emission in both compositions that would follow a similar trend of larger and faster responses in ***p-1A*** than that in ***p-1B***. In this regard, a significant difference was observed in the presence of 10 mM acetic acid. Film ***p-1A*** showed a remarkable case, with a 21% quenching of the total fluorescence at 10 min and a final quenching of 73% at 1 h (Figs. 6b and 7). Film ***p-1B*** displayed a similar behavior, with a more conservative 49% quenching (Figs. 7 and S10). Further experiments revealed that the Limits of Detection (LODs) of the films against stepwise increased concentrations of AcOH also depended on composition. ***p-1A*** achieved a LOD of 1.02 × 10^–3^ M, while ***p-1B*** reached a LOD of 1.70 × 10^–3^ M (Figs. S11 and S12). In this sense, the quantum yields (%Φ) of the systems (Fig. S13) offered slightly different values. %Φ for ***p-1A*** was 0.13, while %Φ for ***p-1B*** was 0.15. Both values were determined in THF solutions, but it remains unclear how these values relate to the overall behavior in the solid state. In addition, competition experiments were performed on the films. In these experiments, pristine specimens were first exposed to the anions individually, and then exposed to AcOH (Fig. S14). This was done to determine whether or not AcOH could displace the former in the solid state and thus, yield a response in a trend compatible with the earlier results. The results showed that the systems mainly responded to the protonated counterpart of acetate.

These results suggest that the interaction between anions and the Zn-Salphen complex can produce either a quenching or an enhancement of the fluorescent emission, depending on several possible reasons that may occur individually or collectively. These reasons include: 1) the capacity of each guest to interact with the binding sites of the Zn-Salphen complex; 2) the ability of each guest to disrupt interchain aggregation that occurs at the domains containing local assemblies of Zn-Salphen; 3) the ability of the films to effectively internalize each guest toward the Zn-Salphen units and to produce host–guest association events in a differential manner, depending on the internal supramolecular environment derived from composition. Given this scenario, it is reasonable to infer that the strongly marked quenching produced by acetic acid is more likely due to a facilitated migration of the neutral guest, in combination with proton exchange pathways that facilitate de-aggregation at the Zn-Salphen domains. However, further studies are needed to either confirm or refute this possibility. For example, such studies could use metal-Salphen counterparts that either remain fluorescent in solid state films via AIEE mechanisms or do not proceed under emission schemes as the latter one, and, simultaneously, exhibit association against guests in a similar manner. However, these studies would fall outside the scope of the present work and are thus not included in the study.

## Conclusions

The fluorescence evaluation of the non-symmetrical Zn-Salphen complex **1** was reported for the first time. In a coordinating solvent, the complex exhibited very low fluorescence. However, in a less coordinating solvent, the emission was significantly enhanced due to the aggregated form of the complex. This magnification of the emission (18 times) in the aggregated state induced by the change in the solubility of the medium is indicative of AIEE behavior of molecule **1**, which is likely due to restricted intramolecular rotation of the phenyl ring attached to the imine group. This finding opens the door for further investigation into the modification of the substituents in this position. Furthermore, the synthesis of new co-polymers was accomplished by radical polymerization of complex **1** and acrylic monomers MMA/*n*-BuA at different compositions (*i.e.* 30:70, 40:60, 50:50, 60:40) through a full factorial design of experiments, from which, functional formulations (MMA/*n*-BuA: 50:50 and 40:60) were selected for further evaluations. The new polymers ***p-1A*** (50:50 MMA:*n*-BuA) and ***p-1B*** (40:60 MMA:*n*-BuA) thus prepared possess excellent physical properties, and their respective films were also easily prepared. Fluorescent measurements of both polymers in solutions of coordinating and non-coordinating solvents showed no significant difference from the isolated complex **1**. However, a large increase of up to 65 times in the photoluminescent emission of the films was found in comparison to their respective solution-based counterparts. The enhanced fluorescence in films was further explored to measure host–guest interactions. The results showed small changes in the emission of the polymers after treatment with guests. The difference in behavior between the guests is hypothesized to be dependent on the capacity of the anion to interact strongly with either the polymer or the Zn-Salphen unit, coordinate the complex, and generate de-aggregation. Finally, a remarkable decrease in fluorescence of more than 73% and 49% for ***p-1A*** and ***p-1B***, respectively, in the presence of acetic acid was found for both films. This opens the possibility for the films to be used as portable, optical and binary (yes/no: turn-off/turn-on) sensing platforms that eliminated the need for expensive measuring devices.

## Experimental

### Materials and Methods

All starting compounds and solvents were purchased from Sigma-Aldrich and used without further purification, unless otherwise stated. Synthesis, purification and spectroscopical confirmation of **1** was carried out according to procedures reported earlier by our group [34]. TGA characterization was carried out under air atmosphere at 10 °C min^−1^ on a Perkin Elmer TGA400 apparatus from 30 °C to 500 °C. DSC data was measured under N_2_ with an increase of 10 °C min^−1^ on Mettler Toledo DSC1 calorimeter at Unidad de Servicios de Apoyo a la Investigación y a la Industria (USAII) of the School of Chemistry-UNAM; conditioning cycles: 1) r.t. to 130 °C, 2) isothermal at 130 °C for 10 min, 3) 130 °C to − 100 °C, 4) isothermal at − 100 °C for 10 min; measurement cycle: 5) − 100 °C to 250 °C. Gel permeation chromatography (GPC) determinations were made on an Agilent Technologies 1290 infinity UHPLC with a Q-TOF detector 6530 DUAL AJ ESI using chromatography-grade tetrahydrofuran as solvent at Unidad de Servicios para la Industria Petrolera (USIP) of the School of Chemistry-UNAM. All nuclear magnetic resonance (NMR) measurements were carried out on a Magritek Spinsolve 80 MHz spectrometer at room temperature, and chemical shifts are given in parts per million vs TMS. UV–Vis absorption measurements were performed in a Dynamica Halo XB-10 UV–Vis apparatus. All fluorescent measurements were performed in a Hinotek F96pro fluorescence spectrophotometer apparatus with an emission filter (λ_exc_ = 365 nm).

### Synthesis and Characterization of Copolymers *p*-1A and *p*-1B

A mixture of Zn-Salphen complex **1**, MMA and *n-*BuA was homogenized. Then, 200 μL of THF, and azobisisobutyronitrile (AIBN) (10 mg/g_total mass_) was added to the monomer mixture. The resulting solution was degassed by bubbling argon into the mixture. The reaction was sonicated at 75 °C in a Sonorex Digitec ultrasonic bath (Bandelin electronic GmbH & Co. KG, Berlin, Germany) during 30 min, then ultrasonic stir was turn off and the reaction was kept at 80 °C during 30 min. Finally, reaction was allowed to reach room temperature.

poly[(Zn-Salphen)_2%_-stat-(*n*-BuA)_49%_-stat-(MMA)_49%_], ***p-1A***. Nominal mass ratio (Zn-Salphen):MMA:*n*-BuA = 2:49:49 (50:50 for MMA:*n*-BuA respectively). T_d_ (TGA): T_5_ = 342 °C, T_10_ = 358 °C, T_o_ = 364 °C, T_f_ = 411 °C, T_p_ = 394 °C (first derivative peak). Average molecular weight (GPC): ${\overline{{\varvec{M}}} }_{{\varvec{n}}}$= 1.8 × 10^5^ g mol^−1^, ${\overline{{\varvec{M}}} }_{{\varvec{w}}}$= 4.0 × 10^5^ g mol^−1^, ${\overline{{\varvec{M}}} }_{{\varvec{v}}}$= 7.0 × 10^5^ g mol^−1^, ${\overline{{\varvec{M}}} }_{{\varvec{z}}}$= 7.7 × 10^5^ g mol^−1^; *Đ*_M_ = 2.17, ${\overline{{\varvec{X}}} }_{{\varvec{n}}}$= 1665, ${\overline{{\varvec{X}}} }_{{\varvec{w}}}$= 3622, ${\overline{{\varvec{X}}} }_{{\varvec{n}}(\mathbf{Z}\mathbf{n}-\mathbf{S}\mathbf{a}\mathbf{l}\mathbf{p}\mathbf{h}\mathbf{e}\mathbf{n})}$= 7.4, ${\overline{{\varvec{X}}} }_{{\varvec{w}}(\mathbf{Z}\mathbf{n}-\mathbf{S}\mathbf{a}\mathbf{l}\mathbf{p}\mathbf{h}\mathbf{e}\mathbf{n})}$= 16.1, ${\overline{{\varvec{X}}} }_{{\varvec{n}}(\mathbf{M}\mathbf{M}\mathbf{A})}$= 538, ${\overline{{\varvec{X}}} }_{{\varvec{w}}(\mathbf{M}\mathbf{M}\mathbf{A})}$= 1170 ${\overline{{\varvec{X}}} }_{{\varvec{n}}({\varvec{n}}-\mathbf{B}\mathbf{u}\mathbf{A})}$= 1120, ${\overline{{\varvec{X}}} }_{{\varvec{w}}({\varvec{n}}-\mathbf{B}\mathbf{u}\mathbf{A})}$= 2436. T_g_ (DSC): 21.3 °C, ΔCp normalized to sample weight and number-average molecular weight 5.87 × 10^4^ J mol^−1^ K^−1^. FT-IR (ATR)/cm^−1^: 2954, 2875 (CH_2_), 1726 (C = O), 1615 (C = N), 1541 (C = C), 1236, 1143 (C-O). Zn-Salphen mass content estimation (^1^H NMR, CDCl_3_): 2.1%. Data shown in supplementary Figs. S15–S18.

poly[(Zn-Salphen)_2%_-stat-(*n*-BuA)_58.8%_-stat-(MMA)_39.2%_], ***p-1B***. Nominal mass ratio (Zn-Salphen):MMA:*n*-BuA = 2:39.2:58.8 (40:60 for MMA:*n*-BuA respectively). T_d_ (TGA): T_5_ = 318 °C, T_10_ = 352 °C, T_o_ = 362 °C, T_f_ = 410 °C, T_p_ = 393 °C (first derivative peak). Average molecular weight (GPC): ${\overline{{\varvec{M}}} }_{{\varvec{n}}}$= 1.6 × 10^5^ g mol^−1^, ${\overline{{\varvec{M}}} }_{{\varvec{w}}}$= 3.3 × 10^5^ g mol^−1^, ${\overline{{\varvec{M}}} }_{{\varvec{v}}}$= 5.2 × 10^5^ g mol^−1^, ${\overline{{\varvec{M}}} }_{{\varvec{z}}}$= 5.6 × 10^5^ g mol^−1^; *Đ*_M_ = 2.0, ${\overline{{\varvec{X}}} }_{{\varvec{n}}}$= 1488, ${\overline{{\varvec{X}}} }_{{\varvec{w}}}$= 2985, ${\overline{{\varvec{X}}} }_{{\varvec{n}}(\mathbf{Z}\mathbf{n}-\mathbf{S}\mathbf{a}\mathbf{l}\mathbf{p}\mathbf{h}\mathbf{e}\mathbf{n})}$= 6.6, ${\overline{{\varvec{X}}} }_{{\varvec{w}}(\mathbf{Z}\mathbf{n}-\mathbf{S}\mathbf{a}\mathbf{l}\mathbf{p}\mathbf{h}\mathbf{e}\mathbf{n})}$= 13.2, ${\overline{{\varvec{X}}} }_{{\varvec{n}}(\mathbf{M}\mathbf{M}\mathbf{A})}$= 481, ${\overline{{\varvec{X}}} }_{{\varvec{w}}(\mathbf{M}\mathbf{M}\mathbf{A})}$= 964 ${\overline{{\varvec{X}}} }_{{\varvec{n}}({\varvec{n}}-\mathbf{B}\mathbf{u}\mathbf{A})}$= 1001, ${\overline{{\varvec{X}}} }_{{\varvec{w}}({\varvec{n}}-\mathbf{B}\mathbf{u}\mathbf{A})}$= 2008. T_g_ (DSC): 34.3 °C, ΔCp normalized to sample weight and number-average molecular weight 5.11 × 10^4^ J mol^−1^ K^−1^. FT-IR (ATR)/cm^−1^: 2952, 2876 (CH_2_), 1725 (C = O), 1615 (C = N), 1510 (C = C), 1237, 1143 (C-O). Zn-Salphen mass content estimation (^1^H NMR, CDCl_3_): 2.6%. Data shown in supplementary Figs. S19–S22.

### Spectroscopic and Fluorescence Measurements

Study with Zn-Salphen complex **1**. Measurements of the UV–Vis absorption and fluorescent spectra in solution were achieved at final concentration of 5.04 × 10^–5^ M in THF, chloroform or the mixture THF:H_2_O. Spectra of polymers ***p-1A*** and ***p-1B*** in solution were obtained from 0.5 g/mL solutions of the respective polymer in THF or CHCl_3_. Film spectra were obtained by direct measure of absorption or fluorescent emission using special settings for solid state specimens.

AIEE study of complex **1**. A 1.01 × 10^–3^ M stock solution of **1** in THF was prepared. Then, 2 mL of a solution with a final concentration of 5.04 × 10^–5^ M was prepared, by adding 100 μL of the stock solution and the corresponding fraction of THF, followed by a dropwise addition of the corresponding fraction of water under stirring. Immediately, the fluorescence spectrum was acquired in the range between 390–700 nm.

Fluorescence sensing of films ***p-1A*** and ***p-1B***. Solutions of the corresponding guest at concentration 0.01 M were prepared using deionized water (> 18.2 MΩ cm) and the respective sodium or potassium salts: SCN^−^, OAc^−^, Cl^−^ and Br^−^ from potassium, F^−^ from sodium. In a typical experiment, polymer strip film (*ca*. 1.0 cm × 2.5 cm) was fixed inside the quartz cuvette using the setting for solid state specimens, then 3 mL of the corresponding analyte (anion guest or acetic acid) was poured inside the cuvette. Then, fluorescent emission was repeatedly measured every 10 min from 390 to 700 nm.

Quantum yield determinations were carried out under compatible conditions, similar to those described for Fig. 4; for solubility reasons, they were done in THF in the case of the polymer samples, while methanol was employed for the reference Rhodamine B. These experiments were done using 365 nm as excitation wavelength and fluorescent integration was achieved for the ranges 430 nm to 680 nm for the reference and between 530 and 680 nm for the polymer samples to avoid biased analyses due to tailing trends.

### Supplementary Information

Below is the link to the electronic supplementary material.

Supplementary file1 (DOCX 3110 KB)

## Data Availability

Data and material information is provided and will be shared on request.
