# Supplementary material for: Zn-Salphen Acrylic Films Powered by Aggregation-Induced Enhanced Emission for Sensing Applications
Source: J Fluoresc. 2023 Sep 4;34(4):1903–12. doi: 10.1007/s10895-023-03399-6 (PMC11249402; doi:10.1007/s10895-023-03399-6)
Supplement: Supplementary file 1 — Supplementary file1 (DOCX 3110 KB) [file 10895_2023_3399_MOESM1_ESM.docx]

**Supplementary Information for:**

**Zn-Salphen Acrylic Films Powered by Aggregation-Induced Enhanced Emission for Sensing Applications**

***Journal of Fluorescence***

Ernesto Enríquez-Palacios,* Ana Victoria Robledo-Patiño and Gustavo A. Zelada-Guillén.*

School of Chemistry, National Autonomous University of Mexico (UNAM), Circuito Escolar s/n, Ciudad Universitaria, Mexico City 04510, Mexico.

***Correspondence**: e.enriquezpalacios@quimica.unam.mx (E.E.-P.), g.zelada@unam.mx (G.A.Z.-G.)

**Table of content:**

| Page | Figure |  |
| --- | --- | --- |
| S2 | S1 | Normalized emission spectra (λ_exc_= 365 nm) of film ***p*-1A** measured at different times in presence of SCN^-^ (10 mM). |
| S3 | S2 | Normalized emission spectra (λ_exc_= 365 nm) of film ***p*-1A** measured at different times in presence of Cl^-^ (10 mM). |
| S4 | S3 | Normalized emission spectra (λ_exc_= 365 nm) of film ***p*-1A** measured at different times in presence of Br^-^ (10 mM). |
| S5 | S4 | Normalized emission spectra (λ_exc_= 365 nm) of film ***p*-1A** measured at different times in presence of OAc^-^ (10 mM). |
| S6 | S5 | Normalized emission spectra (λ_exc_= 365 nm) of film ***p*-1B** measured at different times in presence of Cl^-^ (10 mM). |
| S7 | S6 | Normalized emission spectra (λ_exc_= 365 nm) of film ***p*-1B** measured at different times in presence of Br^-^ (10 mM). |
| S8 | S7 | Normalized emission spectra (λ_exc_= 365 nm) of film ***p*-1B** measured at different times in presence of SCN^-^ (10 mM). |
| S9 | S8 | Normalized emission spectra (λ_exc_= 365 nm) of film ***p*-1B** measured at different times in presence of F^-^ (10 mM). |
| S10 | S9 | Normalized emission spectra (λ_exc_= 365 nm) of film ***p*-1B** measured at different times in presence of OAc^-^ (10 mM). |
| S11 | S10 | Normalized emission spectra (λ_exc_= 365 nm) of film ***p*-1B** measured at different times in presence of acetic acid (10 mM). |
| S12 | S11 | Assessment of the Limit of Detection (LOD) of a ***p*-1A** film for AcOH. |
| S13 | S12 | Assessment of the LOD of a ***p*-1B** film for AcOH. |
| S14 | S13 | Calibration curves for quantum yield estimation: Rhodamine B, ***p*-1A** and ***p*-1B**. |
| S15 | S14 | Competition experiments of ***p*-1A** and ***p*-1B** with AcOH (10 mM) in the presence of other ions (10 mM) in films. |
| S16 | S15 | ^1^H NMR spectrum of ***p*-1A** in CDCl_3_ at 298 K. |
| S17 | S16 | FT-IR spectrum for ***p*-1A**. |
| S18 | S17 | DSC curve for ***p*-1A**. |
| S19 | S18 | TGA curve for ***p*-1A**. |
| S20 | S19 | ^1^H NMR spectrum of ***p*-1B** in CDCl_3_ at 298 K. |
| S21 | S20 | FT-IR spectrum for ***p*-1B**. |
| S22 | S21 | DSC curve for ***p*-1B**. |
| S23 | S22 | TGA curve for ***p*-1B**. |

**
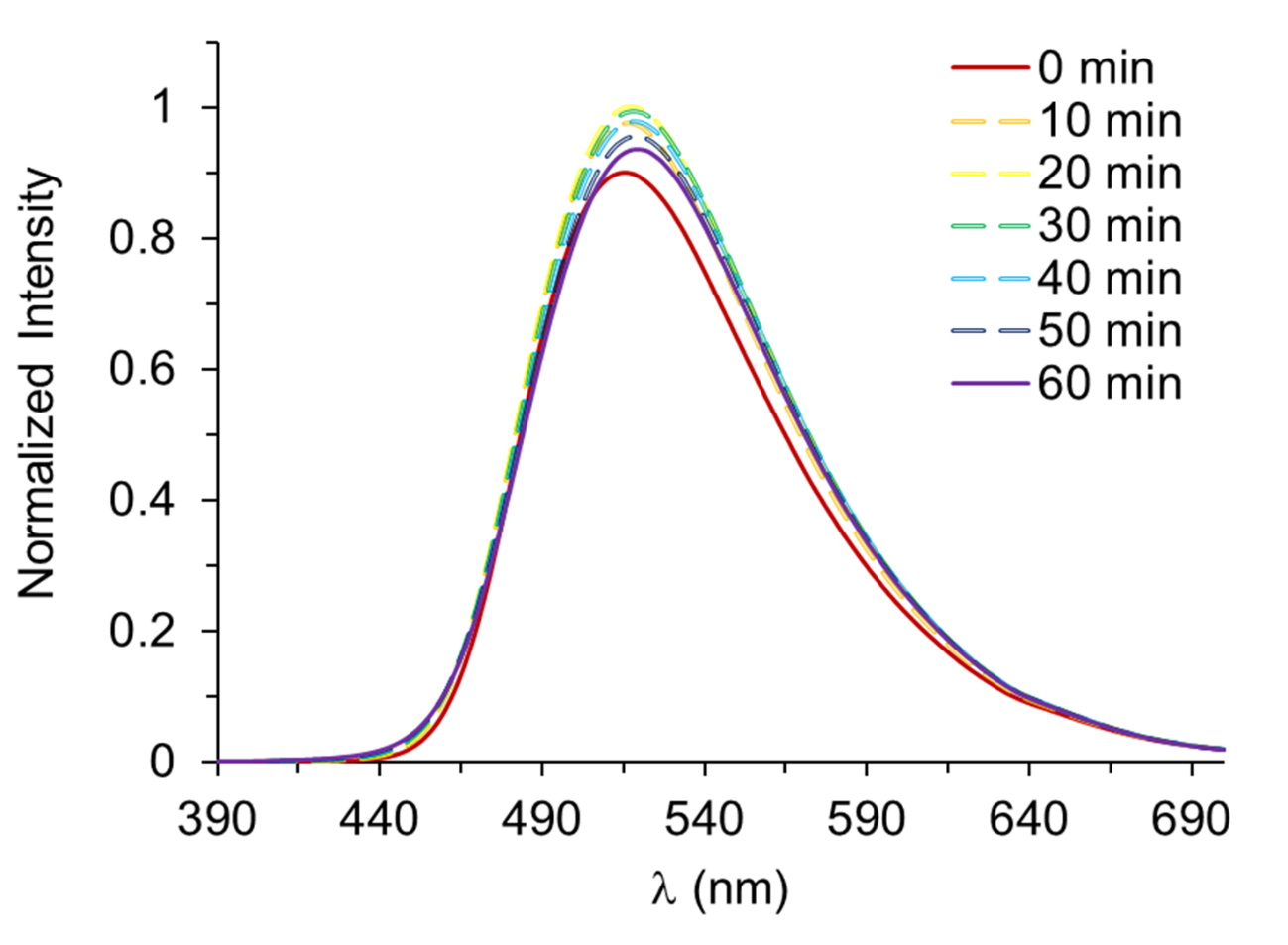
**

**Figure S1.** Normalized emission spectra (λ_exc_= 365 nm) of film ***p*-1A** measured at different times in presence of SCN^-^ (10 mM).

**
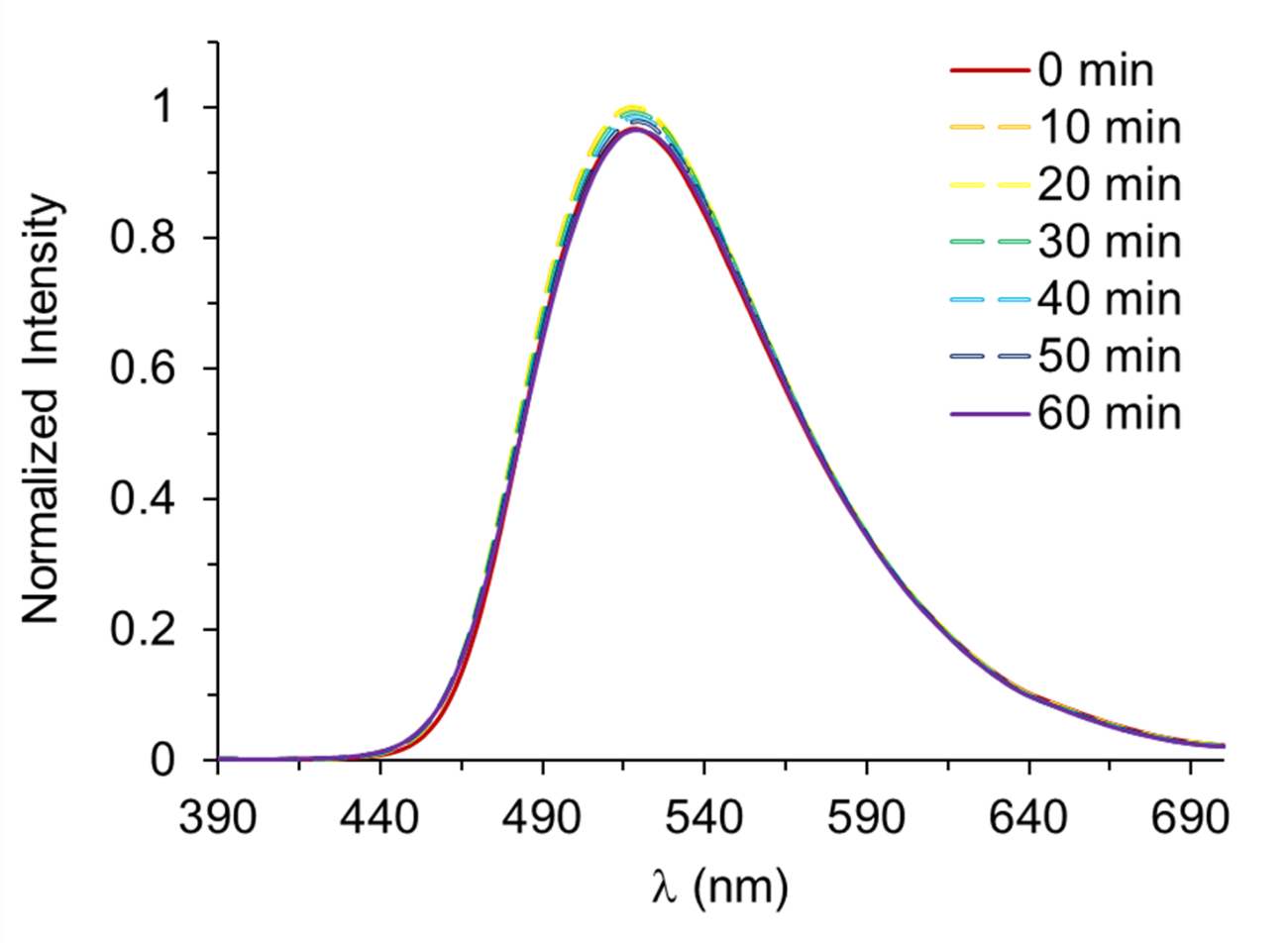
**

**Figure S2.** Normalized emission spectra (λ_exc_= 365 nm) of film ***p*-1A** measured at different times in presence of Cl^-^ (10 mM).

**
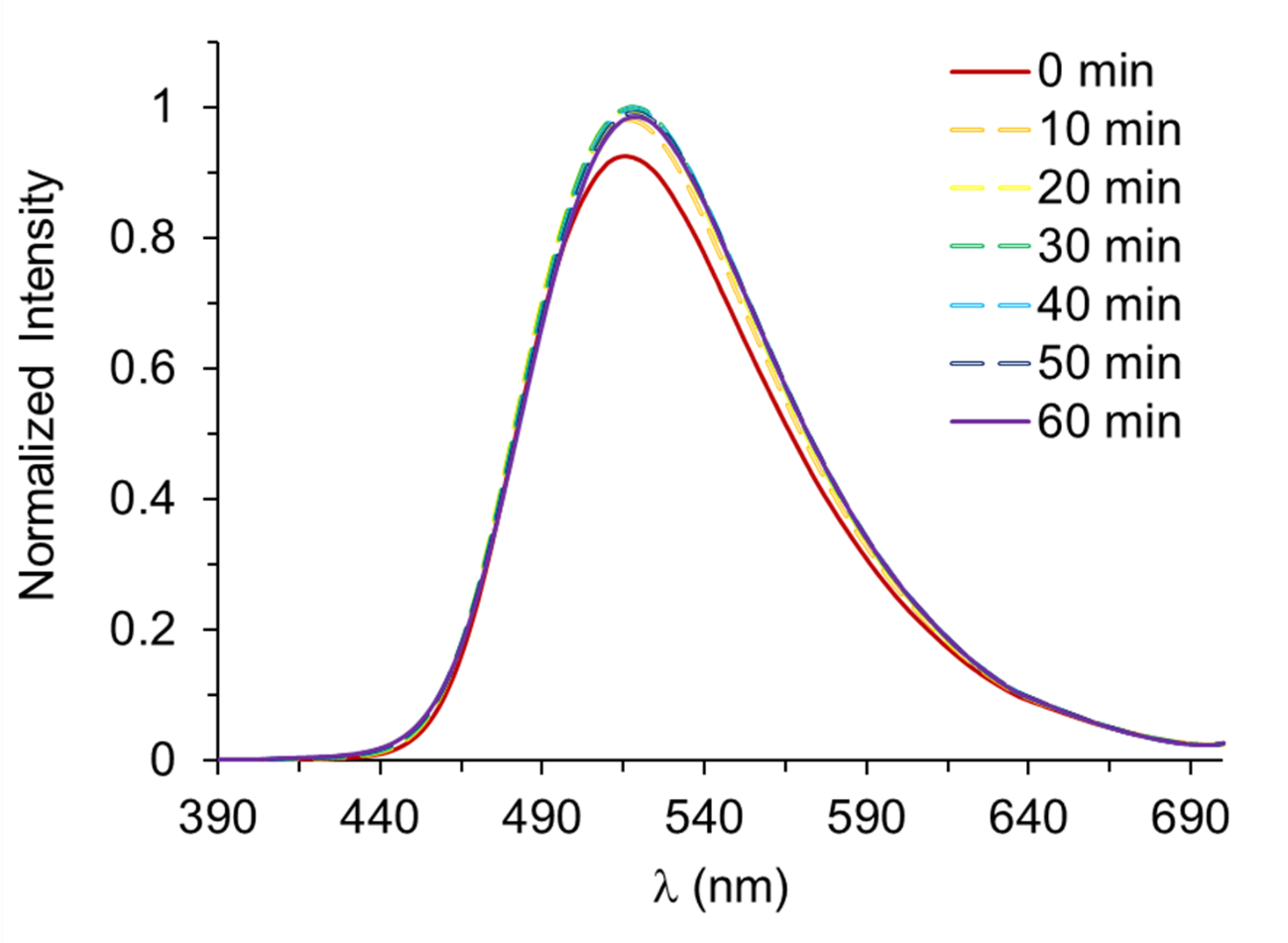
**

**Figure S3.** Normalized emission spectra (λ_exc_= 365 nm) of film ***p*-1A** measured at different times in presence of Br^-^ (10 mM).

**
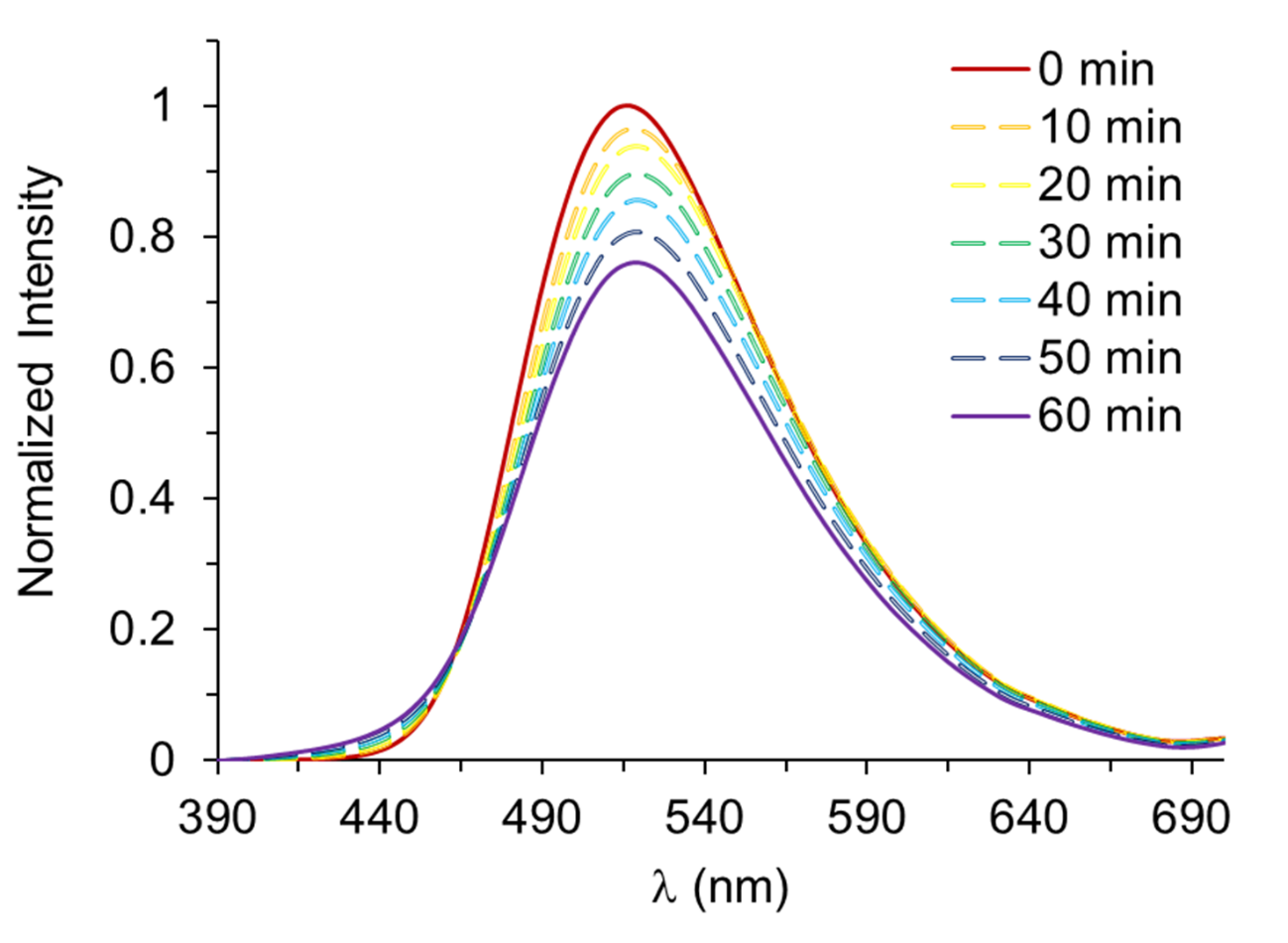
**

**Figure S4.** Normalized emission spectra (λ_exc_= 365 nm) of film ***p*-1A** measured at different times in presence of OAc^-^ (10 mM).

**
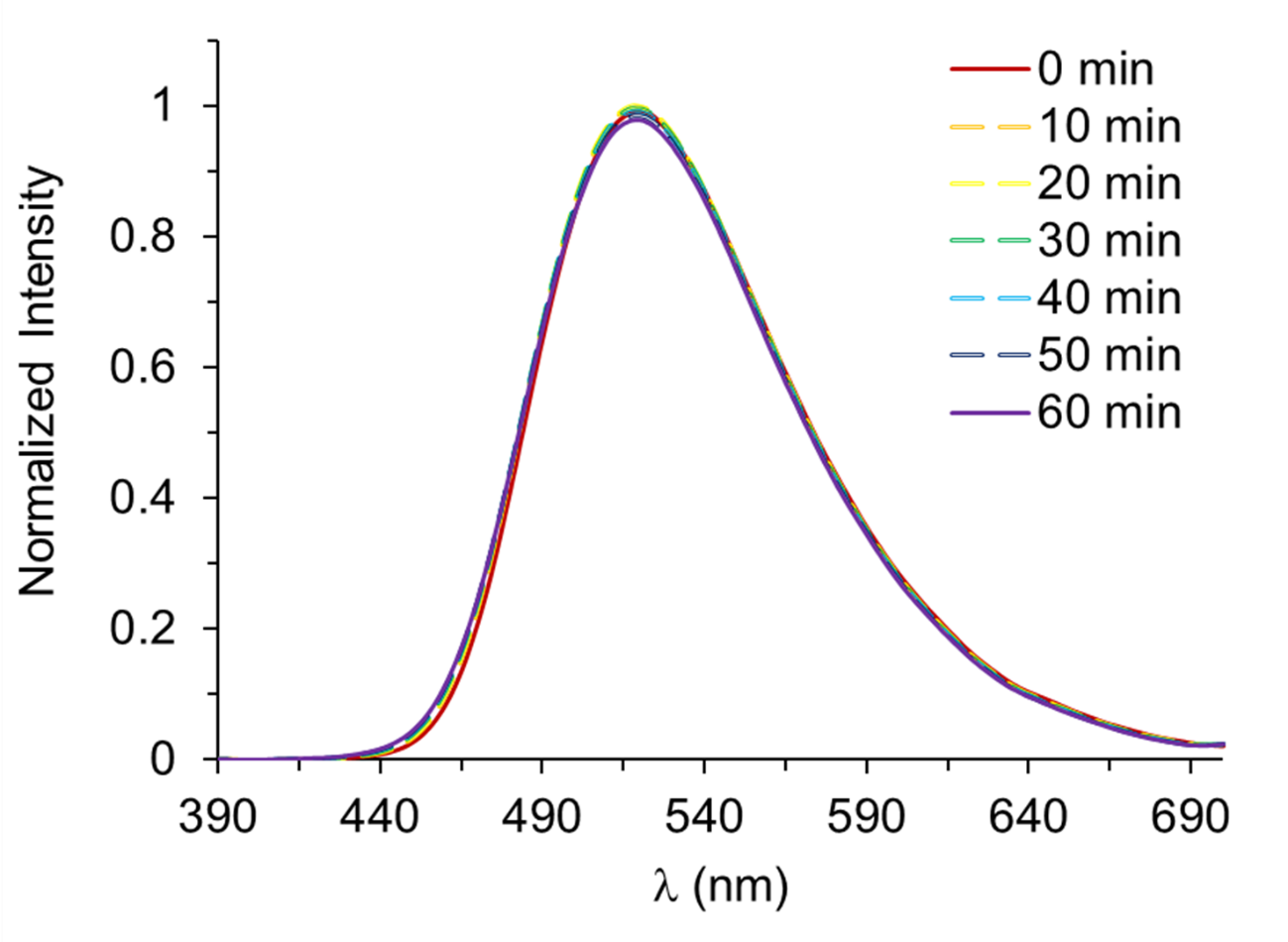
**

**Figure S5.** Normalized emission spectra (λ_exc_= 365 nm) of film ***p*-1B** measured at different times in presence of Cl^-^ (10 mM).

**
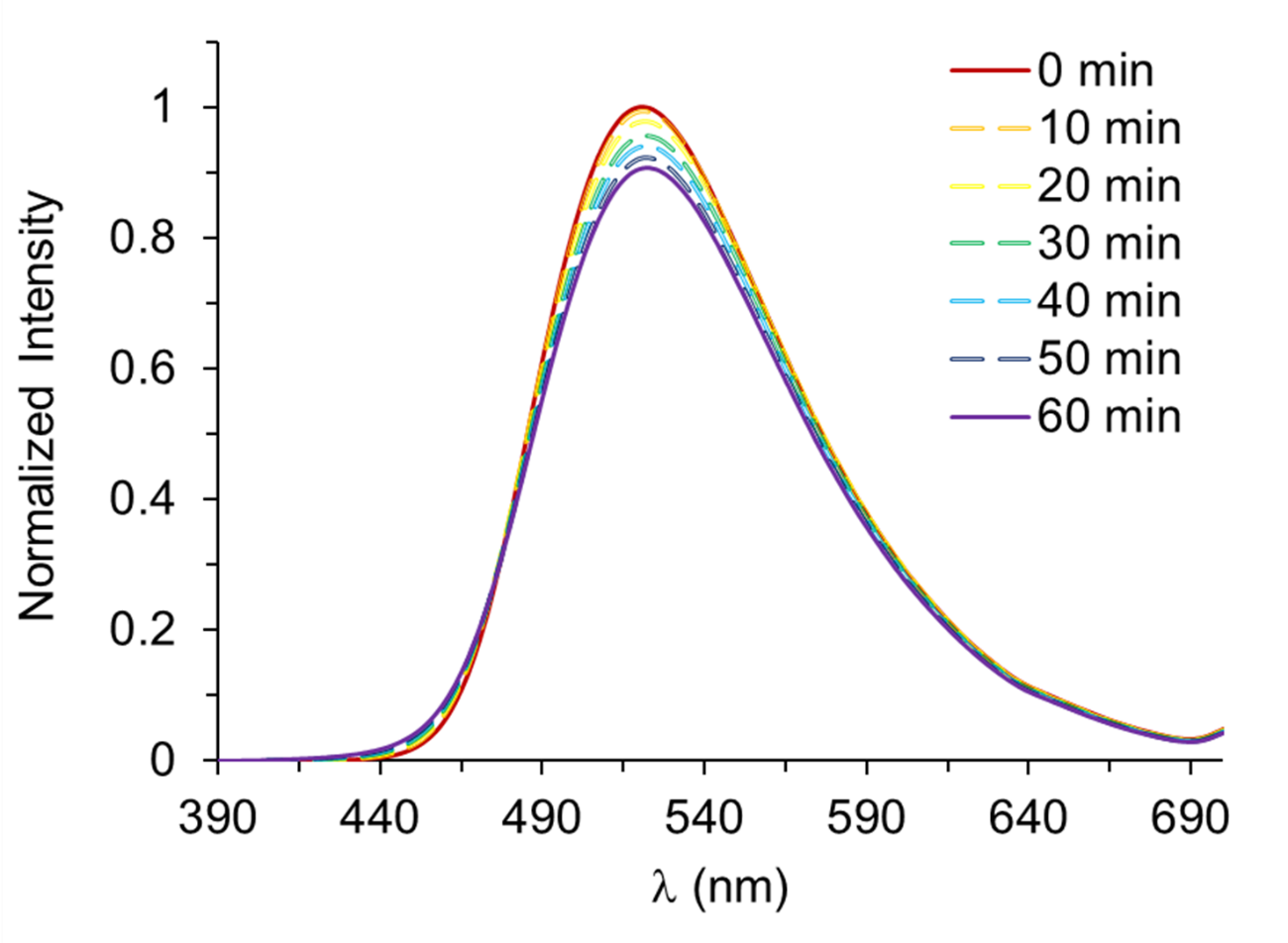
**

**Figure S6.** Normalized emission spectra (λ_exc_= 365 nm) of film ***p*-1B** measured at different times in presence of Br^-^ (10 mM).

**
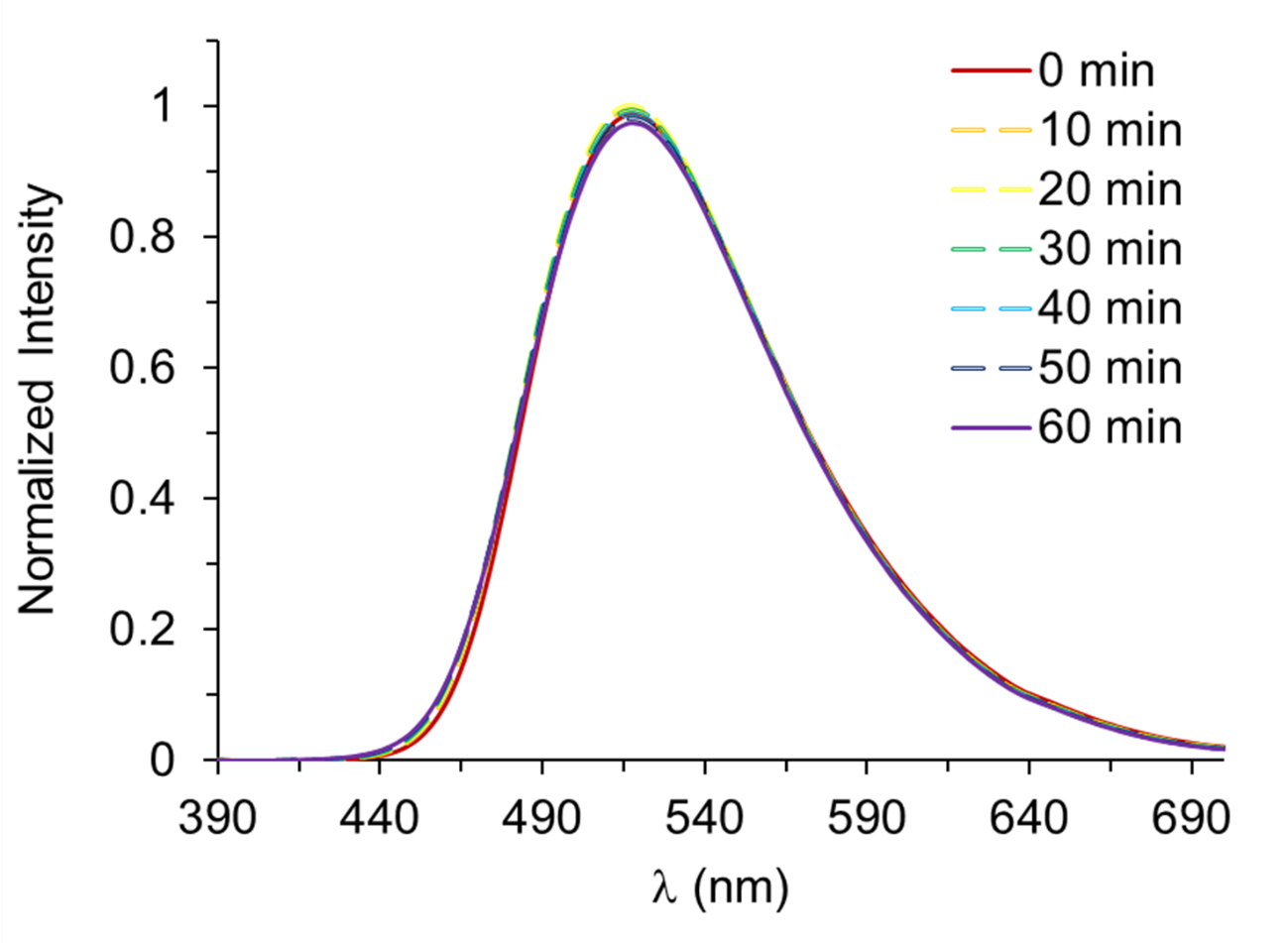
**

**Figure S7.** Normalized emission spectra (λ_exc_= 365 nm) of film ***p*-1B** measured at different times in presence of SCN^-^ (10 mM).

**
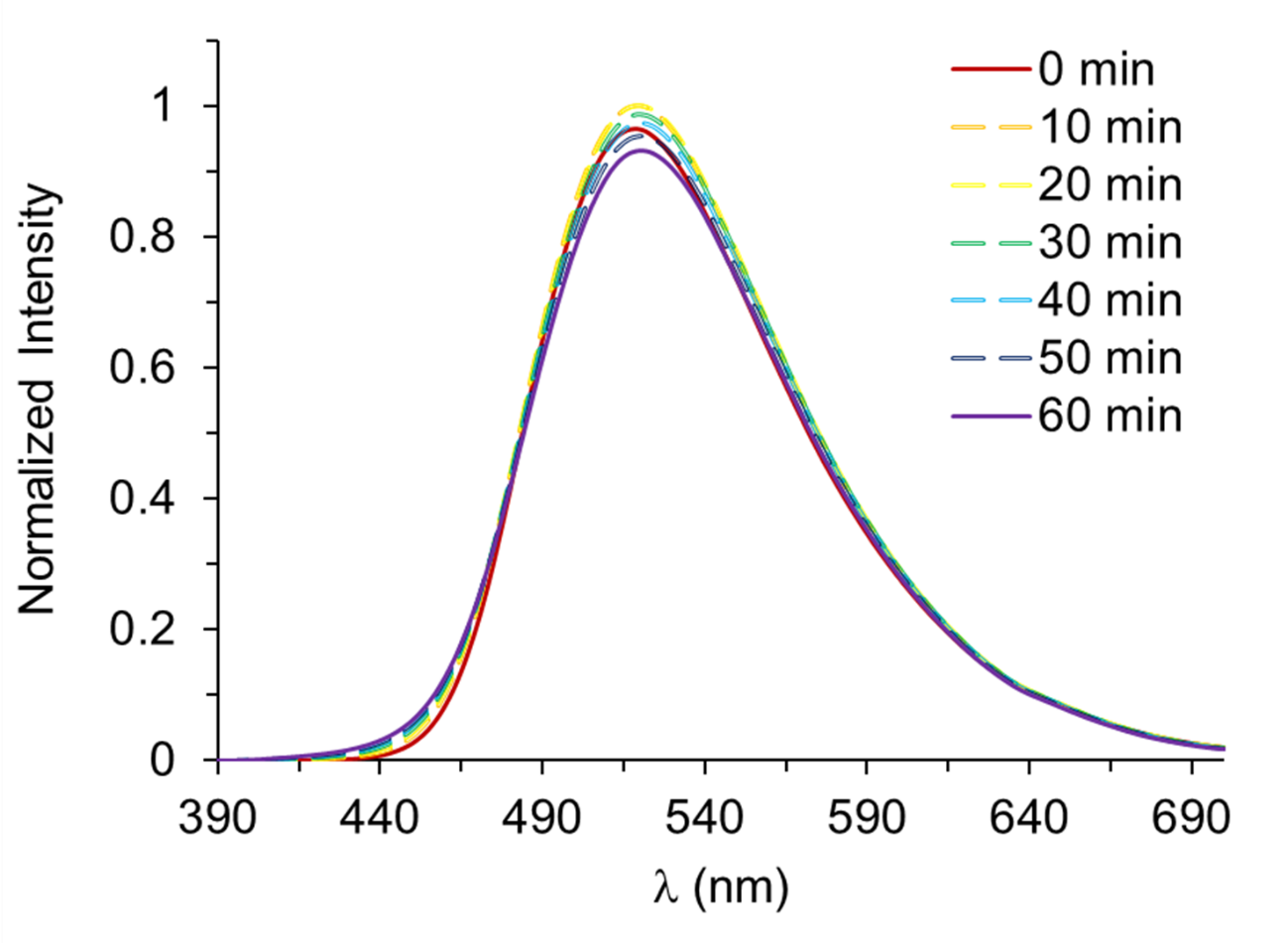
**

**Figure S8.** Normalized emission spectra (λ_exc_= 365 nm) of film ***p*-1B** measured at different times in presence of F^-^ (10 mM).

**
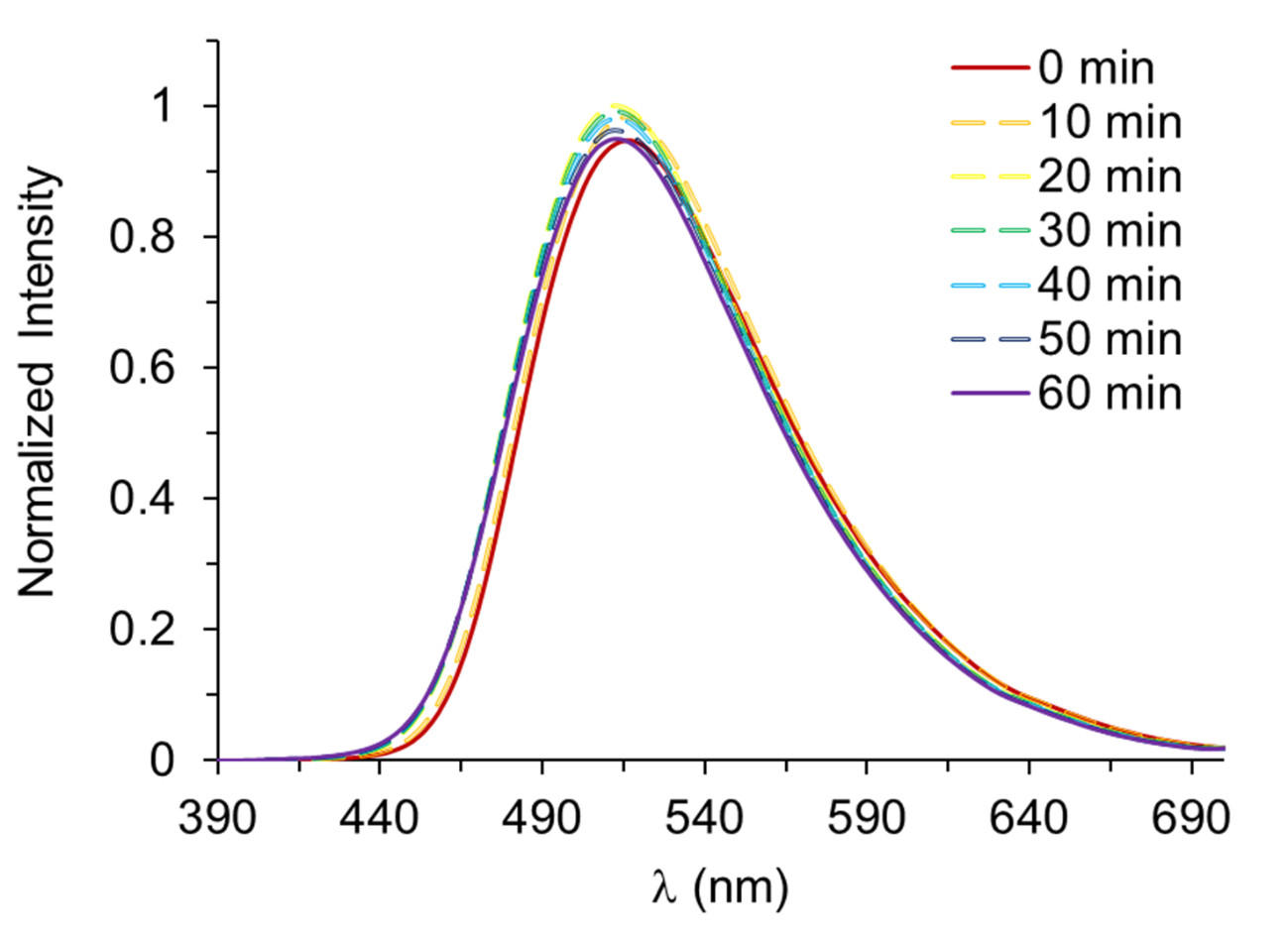
**

**Figure S9.** Normalized emission spectra (λ_exc_= 365 nm) of film ***p*-1B** measured at different times in presence of OAc^-^ (10 mM).

**
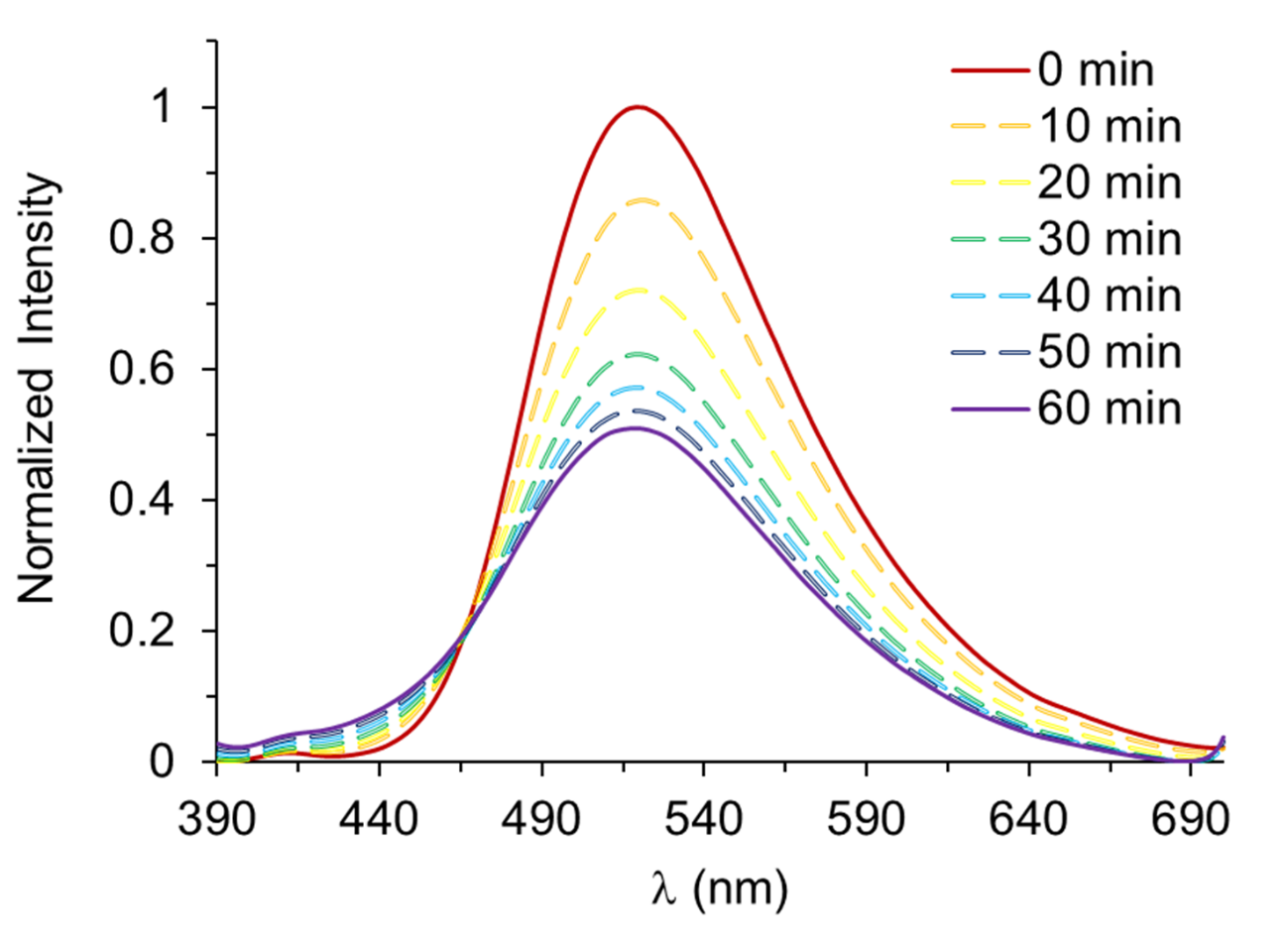
**

**Figure S10.** Normalized emission spectra (λ_exc_= 365 nm) of film ***p*-1B** measured at different times in presence of acetic acid (10 mM).


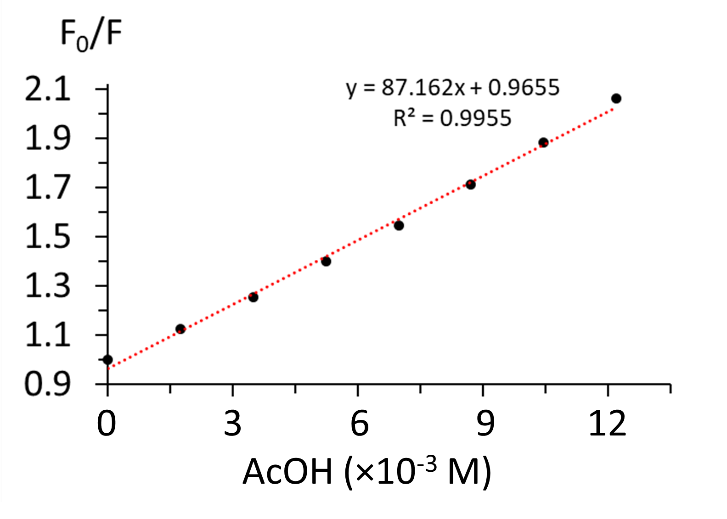


**Figure S11.** Assessment of the Limit of Detection (LOD) of a ***p*-1A** film for AcOH: LOD = 1.02 × 10^-3^ M, Limit of Quantification (LOQ) = 3.08 × 10^-3^ M. Data afforded the next values from linear regression: R^2^ = 0.9955, *m* = 87.162±2.3811 M^-1^, *b* = 0.9655±0.01735, deviation in ordinate = 0.02688, deviations are standard errors.

**
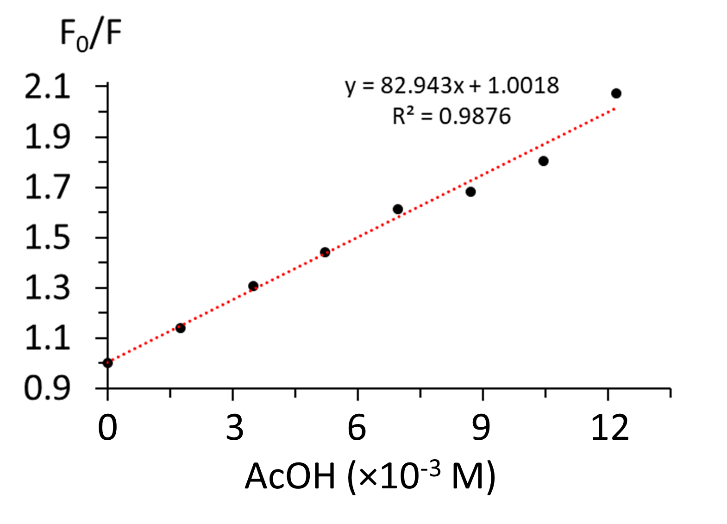
**

**Figure S12.** Assessment of the LOD of a ***p*-1B** film for AcOH: LOD = 1.70 × 10^-3^ M, LOQ = 5.16 × 10^-3^ M. Data afforded the next values from linear regression: R^2^ = 0.9876, *m* = 82.943±3.7955 M^-1^, *b* = 1.0018±0.02765, deviation in ordinate = 0.04284, deviations are standard errors.


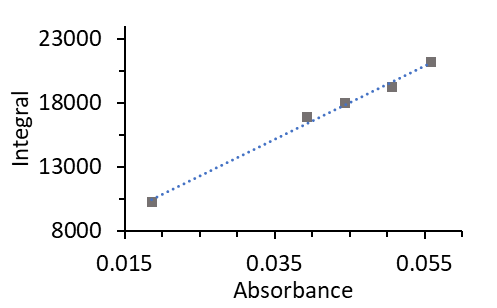

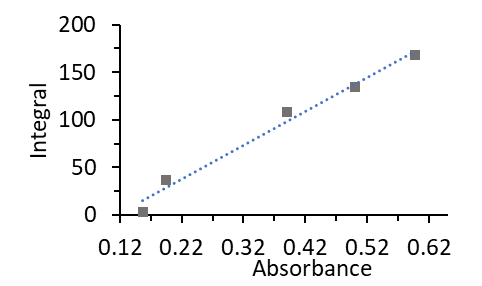

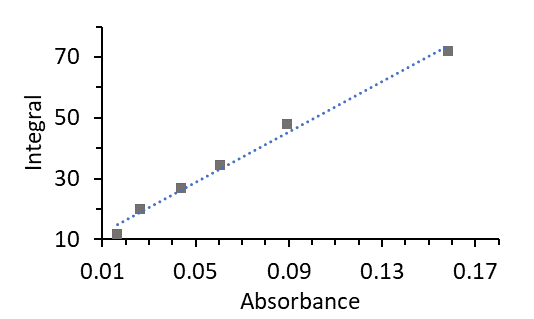


**Figure S13.** Calibration curves for quantum yield estimation: Rhodamine B (left), ***p*-1A** (center) and ***p*-1B** (right). Rhodamine B was used as standard in methanol, evaluated samples ***p*-1A** and ***p*-1B** were measured in THF; estimations were carried out using the equation

$$\Phi_{s}=\Phi_{r}\left( \frac{m_{s}}{m_{r}} \right)\left( \frac{n_{s}}{n_{r}} \right)^{2}$$

where: Φ = fluorescent quantum yield, *m* = gradient of the plot of integrated fluorescent intensity against absorbance, *n* = refractive index of the respective solvent, subscripts “*r*” and “*s*” refer to the reference and evaluated sample respectively. Data afforded the next values from linear regression, not considering significant figures: for Rhodamine, R^2^ = 0.99319, *m* = 286610.432±13705.3633, *b* = 5112.01614±599.19591; for ***p*-1A**, R^2^ = 0.98316, *m* = 357.25039±26.99156, *b* = -41.59072±10.94289; for ***p*-1B,** R^2^ = 0.98955, *m* = 413.53900±21.24499, *b* = 8.26224±1.72716; deviations are standard errors.


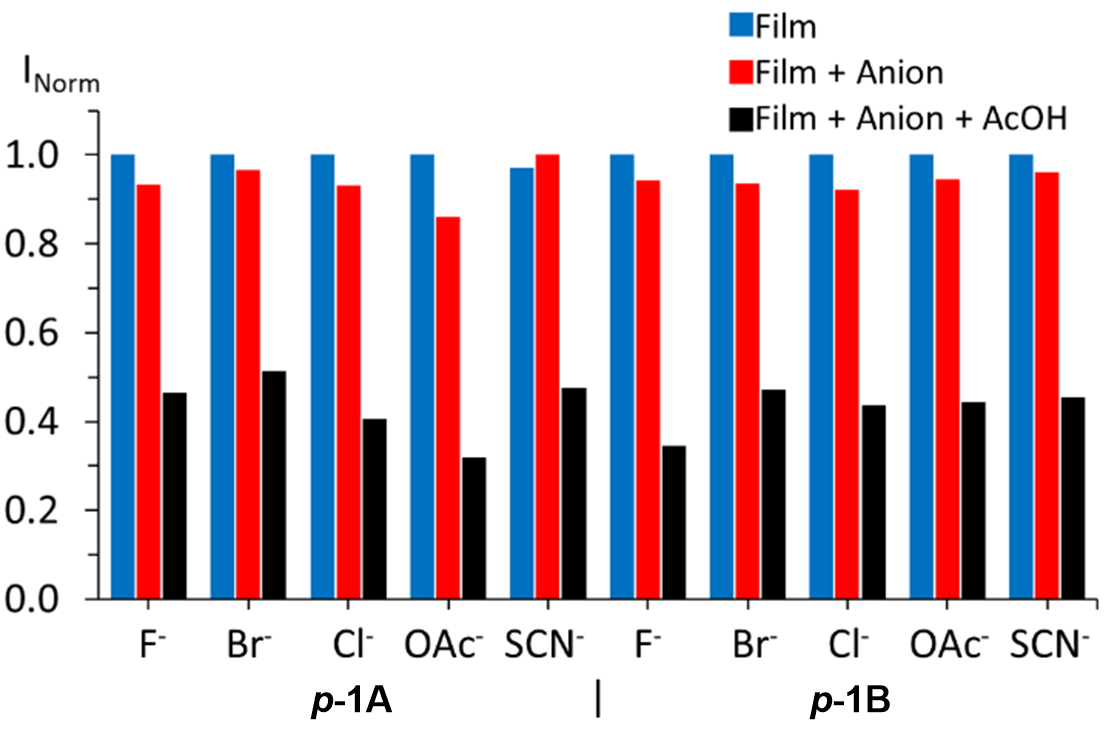


**Figure S14.** Competition experiments of ***p*-1A** and ***p*-1B** with AcOH (10 mM) in the presence of other ions (10 mM) in films. Specimens were incubated for 30 min with each chemical species before measurements.

**Figure S15.** ^1^H NMR spectrum of ***p*-1A** in CDCl_3_ at 298 K.


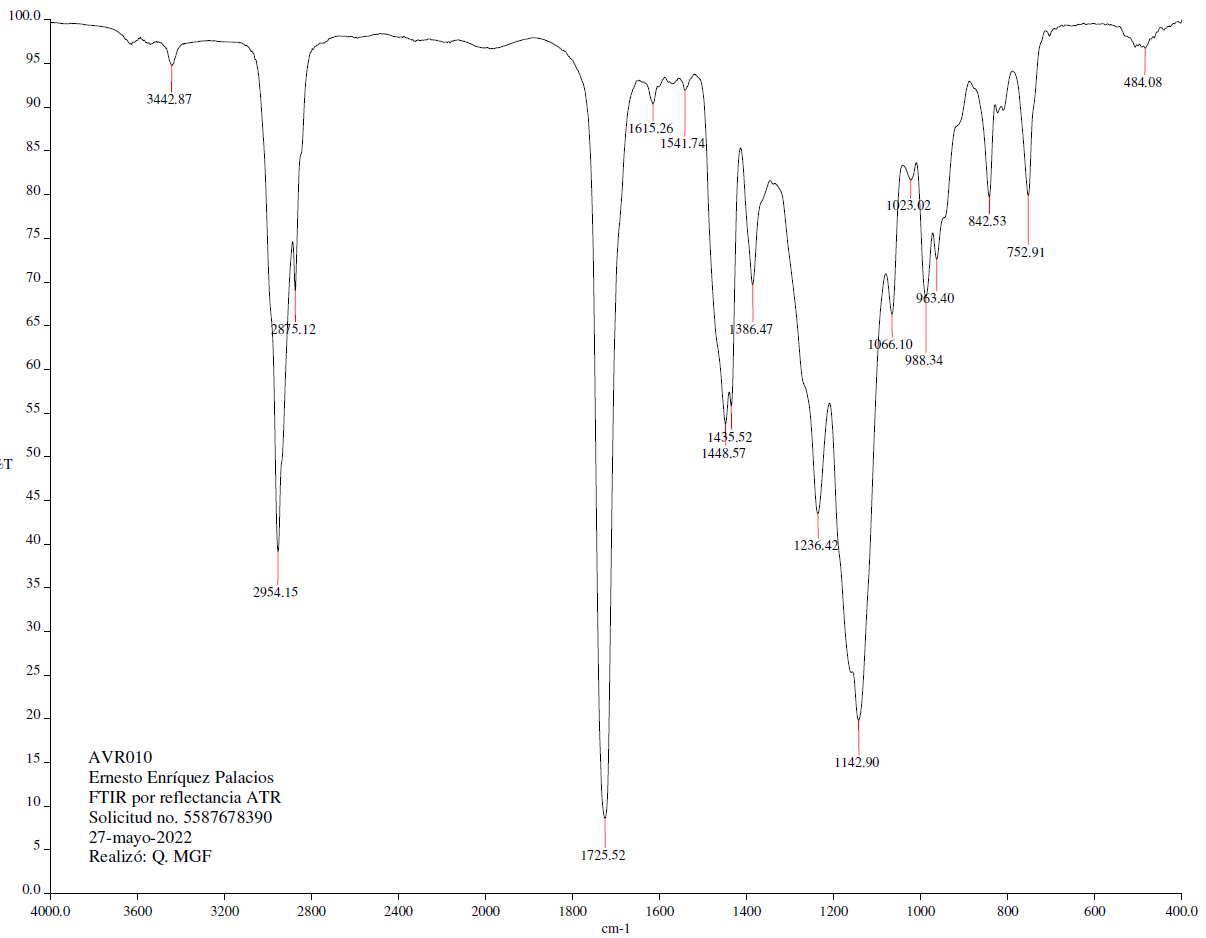


**Figure S16.** FT-IR spectrum for ***p*-1A**.


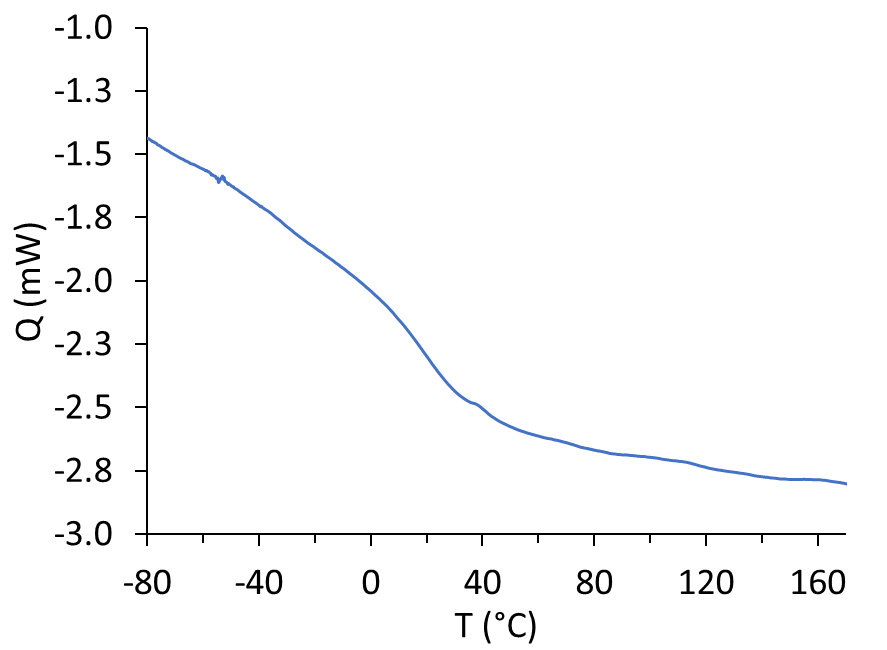


**Figure S17.** DSC curve for ***p*-1A**.


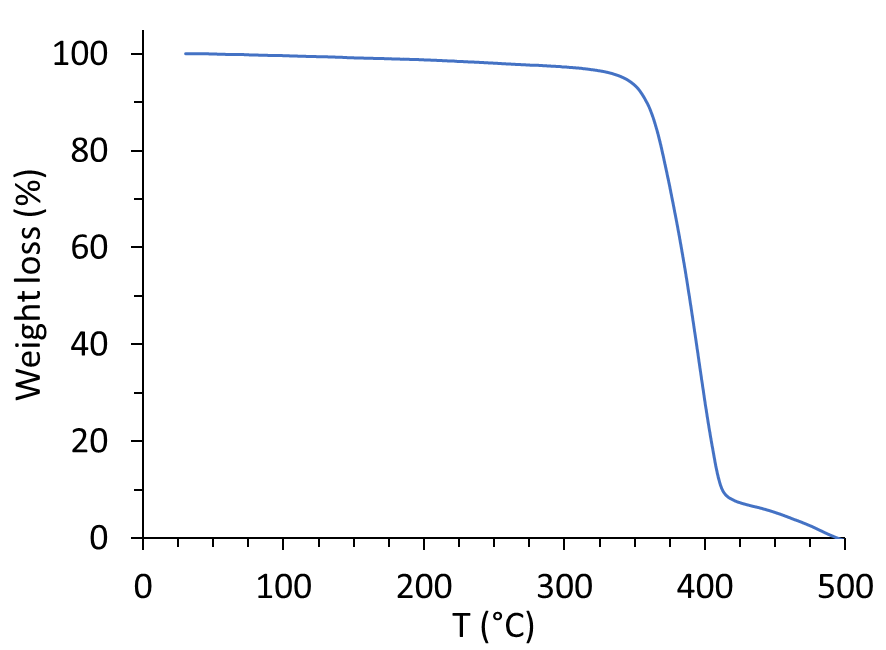
 **Figure S18.** TGA curve for ***p*-1A**.

**Figure S19.** ^1^H NMR spectrum of ***p*-1B** in CDCl_3_ at 298 K.


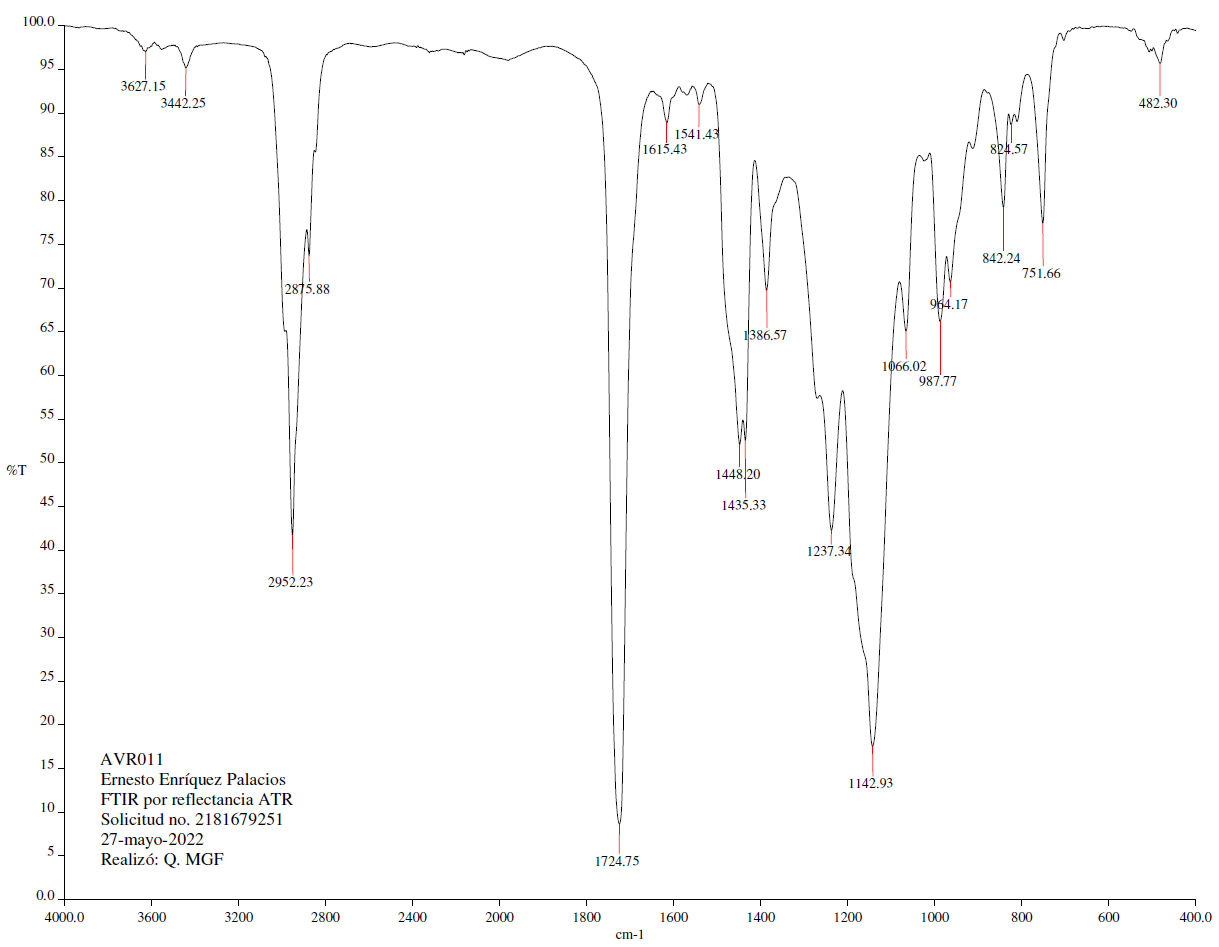


**Figure S20.** FT-IR spectrum for ***p*-1B**.


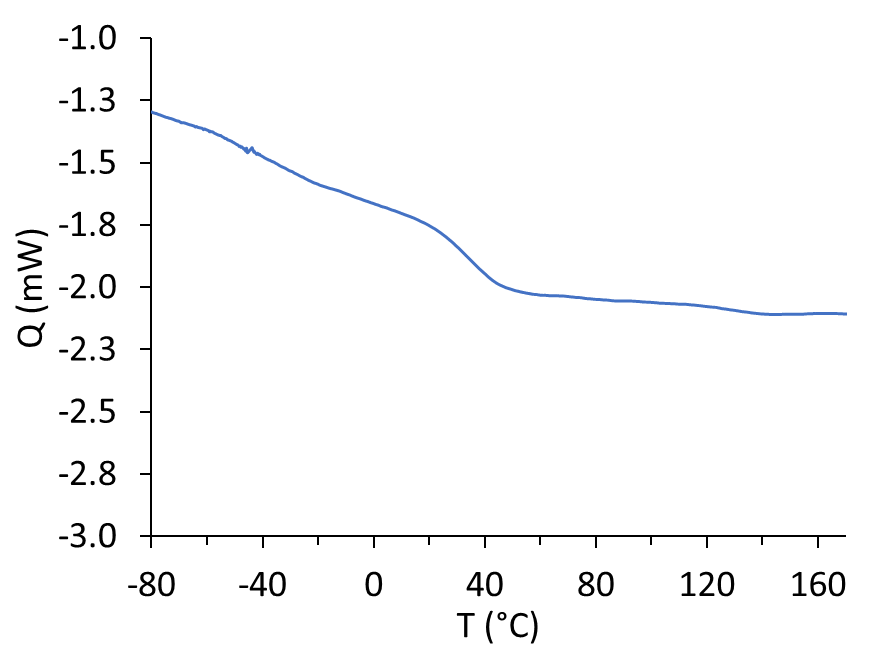


**Figure S21.** DSC curve for ***p*-1B**.


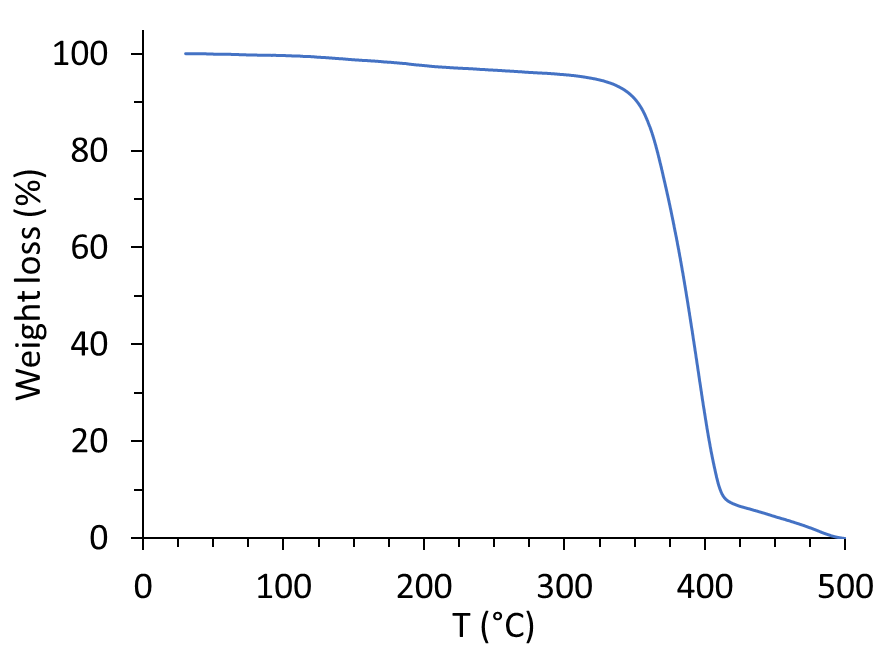


**Figure S22.** TGA curve for ***p*-1B**.
